# The Double-Edged Sword Effect of AI Application Among Clinical Nurses Based on the Job Demands-Resources Model: Qualitative Study

**DOI:** 10.2196/100916

**Published:** 2026-10-02

**Authors:** Xiaoyan Zhang, Jiaxin Fang, Sihan Chen, Jiayin Luo

**Affiliations:** 1 Department of Vascular Surgery, Beijing Hospital, National Center for Gerontology; National Clinical Research Center for Gerontology; The Key Laboratory of Geriatrics of NHC; Institute of Geriatric Medicine, Chinese Academy of Medical Sciences, Beijing, P, Beijing, China; 2 School of Nursing, Beijing University of Chinese Medicine, Beijing, P.R. China, Beijing, China; 3 Department of Outpatient, Beijing Hospital, National Center for Gerontology; National Clinical Research Center for Gerontology; The Key Laboratory of Geriatrics of NHC; Institute of Geriatric Medicine, Chinese Academy of Medical Sciences, No. 1, Dahuajie, Dongdan, Dongcheng District, Beijing, 100730, China, 86 13718677576

**Keywords:** AI, clinical nursing, qualitative research, Job Demands-Resources model, double-edged sword

## Abstract

**Background:**

AI is rapidly transforming clinical nursing, promising administrative relief and decision support. However, the frontline reality presents a double-edged sword effect, where technological empowerment is frequently offset by novel occupational burdens and technostress.

**Objective:**

This study aims to theoretically deconstruct the bidirectional impacts of AI application among clinical nurses and identify buffering conditions, using the Job Demands-Resources (JD-R) theoretical framework.

**Methods:**

A descriptive qualitative study was conducted across multiple general hospitals in mainland China. Using maximum variation and purposive sampling, semistructured in-depth interviews were conducted with registered nurses who actively use clinical AI systems. Data were analyzed using directed qualitative content analysis guided by predefined JD-R constructs.

**Results:**

The analysis revealed 3 overarching domains comprising 9 main themes and 24 subthemes. On the gain path (job resources), AI empowered nurses through a workflow efficiency leap, clinical cognitive empowerment, and professional capital appreciation. Conversely, along the drain path (job demands), hidden costs were exposed, conceptualized as cognitive impediment, relational attrition, and digital involution driven by performance inflation and competitive perfectionism. The interplay between these pathways was perceived to be buffered by contextual mechanisms, specifically nurses’ proactive coping strategies, professional boundary demarcation, and the provision of a cohesive organizational support architecture.

**Conclusions:**

The impact of AI integration in nursing is not technologically deterministic. While AI provides valuable cognitive and operational support, it concurrently generates novel digital demands. To prevent AI from devolving into an occupational hazard, health care administrators must establish multidimensional life cycle AI governance, cultivate comprehensive AI literacy, and safeguard the irreplaceable humanistic core of clinical care.

## Introduction

The global health care landscape is currently undergoing a digital transformation, driven by the rapid expansion of AI technologies. To address the chronic global shortage of nursing personnel and the increasing complexity of patient care, AI has emerged as a strategic imperative [1,2]. In mainland China, this technological wave is particularly pronounced, propelled by robust national policies aimed at constructing “smart hospitals” [3]. Consequently, clinical nursing practice has witnessed the extensive deployment of diverse AI applications. These range from specialized clinical algorithms—such as big data-driven early warning scores, intelligent workforce scheduling, and clinical decision support systems—to generative AI tools increasingly used for administrative tasks like drafting documentation [4-6]. As the largest professional workforce within the health care ecosystem, clinical nurses are positioned at the forefront of this digital revolution, inevitably serving as the core end users of these multifaceted AI systems [7,8].

In line with the optimistic view that AI alleviates administrative burdens and improves decision-making [9], the actual deployment of AI-based systems has led to measurable reductions in documentation transcription time and costs: outpatient clinic letter completion time fell by nearly 8 days, and transcription savings totaled Aus $16,903 (Aus $1=US $0.71 as of May 27, 2026) [10]. However, frontline nursing reality presents a starkly different narrative. Emerging evidence reveals a complex double-edged sword effect [11,12]. While these metrics underscore AI’s capacity to successfully automate certain routine tasks, its integration has spawned novel occupational hazards. Frontline nurses report experiencing alarm fatigue from hypersensitive predictive models, technostress stemming from complex system maintenance, and a pervasive sense of autonomy deprivation induced by ubiquitous digital surveillance [11-13]. Alarmingly, the unprecedented accessibility of AI tools has implicitly elevated organizational performance expectations, triggering a phenomenon of efficiency inflation and subsequent hyper-competition among peers [14,15]. This juxtaposition of technological promise and clinical reality provokes a critical inquiry: are these advanced algorithms genuinely empowering the nursing workforce or inadvertently entrapping them within a rigid, machine-dictated ecosystem?

To theoretically make sense of this seemingly contradictory clinical phenomenon, the Job Demands-Resources (JD-R) model serves as an optimal analytical lens [16]. The JD-R model posits that all psychosocial work characteristics can be classified into 2 overarching categories: job demands and job resources (Multimedia Appendix 1). Viewed through this framework, AI is not a neutral artifact but an active agent dynamically reshaping nursing work. On the one hand, AI can function as a robust job resource—streamlining workflows or providing cognitive support—thereby initiating a motivational process that enhances clinical efficacy. Conversely, unreliable algorithms or heightened performance expectations can morph AI into a stringent job demand. The resulting cognitive overload and socioemotional labor trigger a health impairment process, ultimately culminating in professional burnout. Thus, the JD-R model provides a nuanced architecture to deconstruct the bidirectional impacts of AI [17,18].

Despite the theoretical use of the JD-R model, a critical review of the literature reveals significant methodological and contextual gaps. To date, research concerning AI in nursing has been predominantly quantitative, frequently relying on technology acceptance models to assess willingness to use or focusing on algorithmic diagnostic accuracy [19,20]. While recent qualitative scholarship has begun examining AI perception among nursing leaders and researchers [6,21], in-depth qualitative inquiries explicitly centered on clinical nurses’ heterogeneous interactions with diverse AI applications remain limited. Standardized questionnaires inherently fail to capture psychological undercurrents, such as the cognitive friction of verifying AI hallucinations or the subtle erosion of human-machine trust [22]. Furthermore, within the uniquely high-intensity health care context of mainland China, the unintended sociocultural consequences of AI integration—specifically the phenomena of efficiency inflation and professional involution—remain largely unexplored.

To bridge this knowledge gap, this study aims to deeply explore the “double-edged sword” effect of AI applications among clinical nurses, using directed qualitative content analysis (DQCA) [23] guided by the JD-R model. The novelty of this study is to theoretically deconstruct the bidirectional impacts of AI on clinical nurses and to identify buffering conditions, using the JD-R theoretical framework. Specifically, this qualitative inquiry has three objectives: (1) explicate the gain path by mapping how AI functions as an empowering job resource; (2) delineate the drain path by identifying how AI simultaneously acts as a novel digital job demand; and (3) uncover the perceived buffering conditions that navigate the interplay between these dual pathways. Ultimately, this study endeavors to provide a robust qualitative empirical foundation for health care organizations to develop more sustainable, human-centric AI integration strategies.

## Methods

### Study Design

A descriptive qualitative study design was adopted to deeply explore the lived experiences of clinical nurses interacting with AI. Given that this study is theoretically anchored in the JD-R model, we used a DQCA approach. DQCA is particularly appropriate when prior theory exists about a phenomenon but would benefit from further description or contextualization. This approach allowed us to deductively map nurses’ experiences onto the predefined JD-R constructs while retaining the inductive flexibility to capture emerging, culturally specific phenomena. This study is reported in accordance with the Standards for Reporting Qualitative Research (SRQR) [24] guidelines.

### Study Setting

#### Study Setting and Participant Context

The study was conducted in mainland China between November 2025 and March 2026. In contemporary Chinese health care, the practice scope of registered clinical nurses is inherently multidimensional, encompassing direct bedside care, clinical administration/quality improvement, and practice-based continuing education and scholarly research. Consequently, frontline nurses interact with a heterogeneous spectrum of AI applications, including embedded clinical AI systems—institutionally integrated, algorithm-driven tools designed for direct patient care, risk monitoring, and operational management (eg, early warning scoring systems and clinical decision support systems)—and generative AI tools driven by large language models (LLMs; eg, DeepSeek [Hangzhou DeepSeek Artificial Intelligence Basic Technology Research Co, Ltd], Doubao [Beijing Chuntian Zhiyun Technology Co, Ltd], or ChatGPT [OpenAI]). Participants were recruited across 8 general hospitals (tertiary and secondary) and 1 affiliated primary community health center in 4 municipalities/provinces (Beijing, Shandong, Guangdong, and Sichuan). The community facility was included as an integrated primary-care arm of a regional tertiary health alliance to capture grassroots AI adoption. In this study, clinical nurses were defined broadly as registered nurses providing direct patient care, including those working in primary care settings, to reflect the full spectrum of AI deployment across different tiers of the health care system.

#### Participant Eligibility, Sampling, and Recruitment

A purposive sampling strategy framed by a maximum variation matrix was adopted to ensure broad coverage across key demographic and clinical dimensions. Inclusion criteria were as follows: (1) registered nurses holding a valid license; (2) currently engaged in direct clinical care with at least 1 year of clinical experience; and (3) having active, hands-on experience with at least one AI-based system—such as embedded clinical AI systems or generative AI tools—in their daily workflow for ≥3 months. Exclusion criteria included nurses exclusively in administrative roles without direct AI clinical application. Participants were recruited via professional nursing association networks and hospital department announcements across participating institutions. A total of 37 eligible nurses were approached; 2 declined due to clinical shift conflicts and 1 withdrew before the interview for personal reasons, yielding a final sample of 34 (completion rate: 91.9%) participants. To construct the sampling matrix, we initially recruited nurses varying in age, professional titles, educational backgrounds, and hospital tiers, aiming to capture the widest possible spectrum of routine AI interaction experiences. Building upon this maximum-variation framework, we then deliberately applied intensified sampling to 2 subgroups of particular theoretical interest: tertiary hospital nurses (n=26, 76.5%) and male nurses (n=13, 38.2%). Tertiary teaching hospitals serve as the primary innovation hubs for advanced AI deployment in China, and oversampling this group allowed us to capture dense, firsthand accounts of complex AI system integration. Similarly, male nurses—a demographic minority within the profession—were intentionally oversampled to ensure that their perspectives on technostress and human-AI interaction were not marginalized in the final analysis. This layered sampling design prioritizes both the breadth of routine experiences and the depth of theoretically critical cases. Enrollment continued until data saturation was achieved, defined as the point where no new codes or themes emerged from the last 3 consecutive interviews.

#### Procedures

Interviews were conducted by 2 qualitative researchers (XZ and JF) trained in advanced qualitative content analysis. Neither interviewer had prior personal, clinical, or administrative relationships with the participants. To ensure reflexivity, both interviewers maintained reflexive journals throughout the study to acknowledge, document, and bracket preexisting assumptions regarding the JD-R model (the detailed interview guide is provided in Multimedia Appendix 2). A total of 14 interviews were conducted face-to-face in private, quiet hospital conference rooms, while 20 were conducted via a secure online video conferencing platform (Tencent Meeting [Tencent]) to accommodate geographically distant participants across provinces. All interviews were conducted in Mandarin Chinese and lasted between 20 and 60 minutes. Field notes were recorded during and immediately after each interview to capture nonverbal cues, vocal hesitations, and emotional responses, which were subsequently integrated into transcript familiarization.

#### Analysis

Audio recordings were transcribed verbatim in Mandarin within 48 hours. Selected verbatim quotes were translated into English by a bilingual nursing researcher and back-translated by an independent native English academic editor to maintain conceptual and cross-language equivalence. Data analysis was managed using NVivo 20 software (QSR International) and followed the DQCA systematic steps: (1) preparation and familiarization—2 researchers independently read the transcripts multiple times alongside field notes to achieve deep immersion in the data; (2) deductive coding—initial coding categories were generated deductively from predefined JD-R constructs, and transcripts were coded by assigning text passages to these predefined categories; (3) inductive coding—data that could not be categorized into the initial JD-R model were assigned new codes inductively using explicit rules; for instance, statements describing technology-driven peer hyper-competition and artificially escalated management performance baselines were inductively coded under digital involution. To ensure coding reliability, 2 coders independently coded an initial batch of 6 transcripts; (4) theme refinement, disagreement resolution, and rigor—the research team held regular consensus meetings to compare codes, resolve discrepancies, and abstract the codes into main themes and subthemes. Discrepancies were discussed until 100% agreement was reached. Theoretical saturation was audited through a cumulative codebook; code generation stabilized at Participant 31, and the final 3 consecutive interviews produced zero new codes or subthemes, confirming data saturation.

### Ethical Considerations

Ethical approval for this study was obtained from the Ethics Committee of Beijing Hospital (approval 2024BJYYEC-KY203-02). Before data collection, all prospective participants were provided with a detailed information sheet outlining the study’s purpose, procedures, and their rights. Written informed consent was obtained from each participant before the interviews commenced. Participants were explicitly informed that their participation was completely voluntary and that they had the right to withdraw from the study at any time without penalty. To address ethical and privacy concerns regarding technology use, the research team explicitly inquired about data security practices during the interviews. All participants who used noninstitutional, general-purpose AI tools reported manually executing strict deidentification protocols (eg, stripping patient names, identification numbers, specific dates, and institutional identifiers) prior to any text or data entry. Disclosures of unauthorized or unapproved AI practices were treated with strict research confidentiality, framed nonpunitively, and analyzed as systemic indicators of unmet operational efficiency demands rather than individual misconduct. To strictly protect participant confidentiality and anonymity, all audio recordings and transcripts were stored securely on password-protected devices. Each participant was assigned a pseudonym, and specific identifying details regarding their employment were carefully modified or redacted to ensure they remained unidentifiable in the presentation of the findings.

### Trustworthiness

To ensure methodological rigor, we adhered to Lincoln and Guba’s evaluative criteria: (1) credibility—member checking was performed by returning a summary of the synthesized themes to 5 participants to verify whether the findings accurately reflected their realities. Additionally, peer debriefing was conducted with a senior qualitative researcher to challenge and refine the analytical interpretations. (2) Transferability—thick descriptions of the study setting, participant demographics, and contextual factors (eg, China’s specific clinical environment) were provided to allow readers to assess the applicability of the findings to other contexts. (3) Reliability—a comprehensive audit trail was maintained, documenting all methodological decisions, coding dictionaries, and analytical memos. Two independent coders performed the analysis, and intercoder agreement was continuously negotiated. (4) Confirmability—the researchers practiced continuous reflexivity by keeping a reflexive journal to acknowledge and bracket their preexisting assumptions regarding AI and the JD-R model, ensuring that the findings were deeply grounded in the participants’ narratives rather than the researchers’ biases.

## Results

### Participant Characteristics

A total of 34 clinical nurses from various departments and hospital tiers were recruited through purposive sampling. The demographic profile of the participants is summarized in Multimedia Appendix 3. The participants’ ages ranged from 23 to 43 years (mean age 30.65, SD 5.56 y), and their nursing experience spanned from 1 to 22 years (mean experience 8.12, SD 5.48 y). The sample consisted of 21 (61.8%) females and 13 (38.2%) males. Regarding educational background, 23 (67.6%) participants held an undergraduate degree, 7 (20.6%) held a postgraduate degree, and 4 (11.8%) had a junior college diploma or below. In terms of professional titles, the majority were intermediate-level nurses (n=19, 55.9%), followed by junior-level (n=11, 32.4%) and senior-level nurses (n=4, 11.8%). Participants were drawn from diverse clinical settings, including surgery (n=14, 41.2%), internal medicine (n=12, 35.3%), emergency (n=4, 11.8%), and intensive care units (n=4, 11.8%). Most participants (n=26, 76.5%) were recruited from tertiary hospitals, with the remainder from secondary (n=7, 20.6%) or community (n=1, 2.9%) health care facilities.

Across the sample, nurses interacted with a dual ecosystem of AI technologies, combining hospital-integrated clinical AI systems (eg, early warning scoring systems, clinical decision support systems, risk prediction systems, and smart infusion pumps) with personally accessed generative AI tools (eg, DeepSeek, Doubao, ChatGPT, and Kimi [Moonshot AI]). Hospital-sanctioned clinical AI systems were characterized by high daily workflow integration for direct bedside care following formal institutional onboarding, whereas generative AI tools were predominantly self-taught and used for administrative drafting, literature synthesis, and health education content creation (see Multimedia Appendix 4 for more details).

### Overview of the Findings

#### Thematic Structure of the Findings

Through DQCA guided by the JD-R framework, we identified 3 overarching domains comprising 9 main themes and 24 subthemes that illustrate the complex double-edged sword effect of AI application in clinical nursing (Figure 1). Specifically, the gain path (job resources) demonstrates the empowering potential of AI by facilitating a workflow efficiency leap, providing clinical cognitive empowerment, and fostering professional capital appreciation. Conversely, the drain path (job demands) highlights the hidden and continuous costs of AI integration, conceptualized as perceived cognitive impediment, relational attrition that challenges humanistic empathy, and digital involution driven by performance inflation and competitive pressures. Furthermore, the interplay between these perceived gains and demands is contextualized by key perceived buffering conditions, specifically nurses’ proactive coping strategies, strict professional boundary demarcation, and the presence of a supportive organizational support architecture. The detailed thematic coding tree is presented in Multimedia Appendix 5.

**Figure 1.**
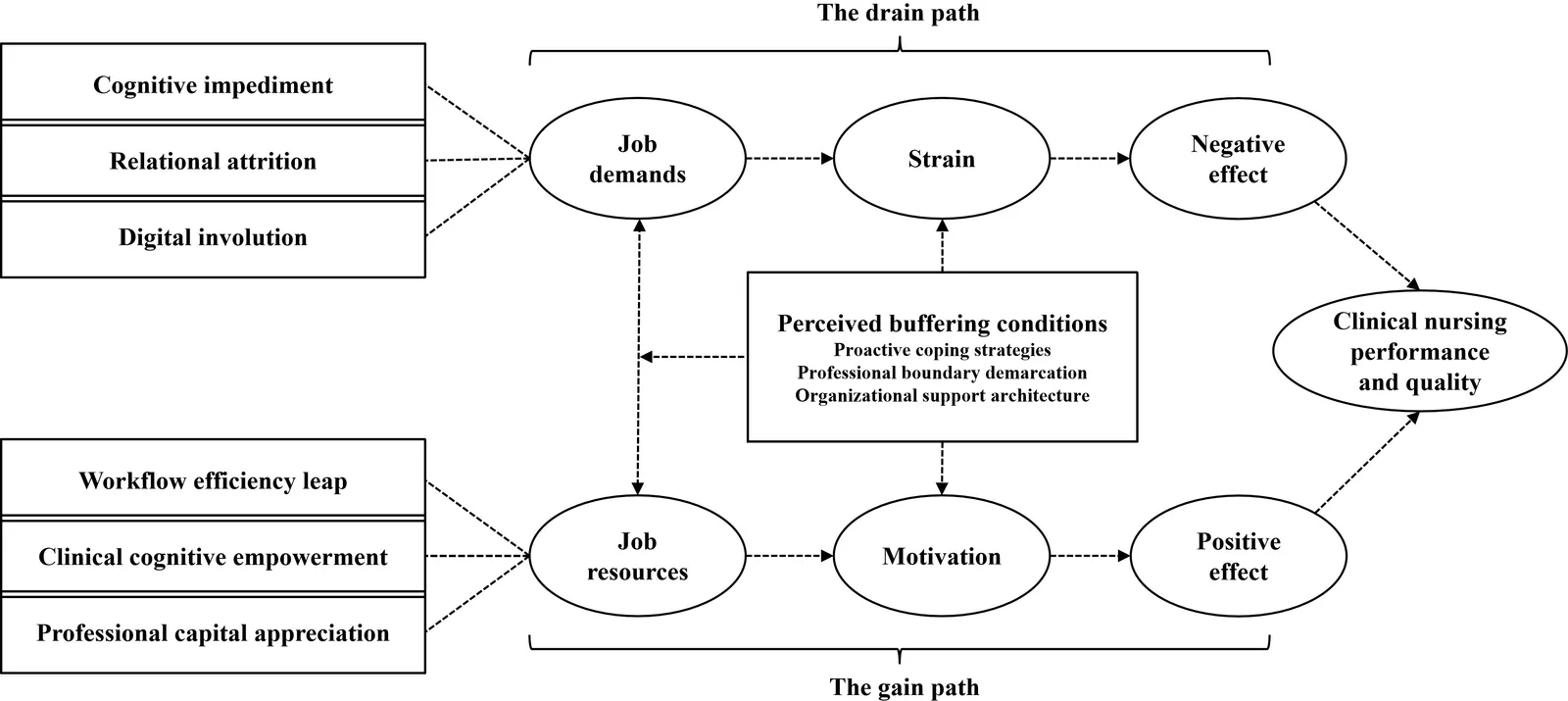
Qualitative conceptual map of the double-edged sword effect of AI application among clinical nurses under the Job Demands-Resources (JD-R) framework.

#### Domain 1: AI as a Job Resource (the Gain Path)

AI emerged as a robust job resource for clinical nurses, triggering a motivational process. By interacting with various AI systems, nurses extracted perceived empowerment, which manifested across 3 distinct themes: workflow efficiency leap, clinical cognitive empowerment, and professional capital appreciation.

##### Theme 1: Workflow Efficiency Leap

In this study, workflow efficiency leap is defined as structural optimization and operational acceleration of daily nursing duties through AI automation, enabling nurses to transition from administrative overextension to streamlined task execution. Participants frequently articulated how AI integration fundamentally remodeled their daily operations across 3 subthemes: documentation automation, task cycle reduction, and team synergy strengthening.

###### Subtheme 1: Documentation Automation

Nurses reported an alleviation of administrative burdens driven primarily by generative AI tools. Rather than expending energy on repetitive typing and formatting, nurses used AI to structure scattered data and polish professional communication. For instance, generative AI tools provided vital support for cross-lingual writing, refining the phrasing of workplace correspondence, and rapidly summarizing lengthy literature. This capability lowered the barrier to content creation and eased the cognitive friction associated with writer’s block. As nurse N10 illustrated: “if I want to create an outline myself, it might take a bit longer. But if AI helps me, it only takes one or two minutes, or even just twenty or thirty seconds” (see Multimedia Appendix 5 for the full dataset).

###### Subtheme 2: Task Cycle Reduction

Both embedded clinical AI systems (eg, automated electronic health record data extraction) and generative AI tools acted as perceived accelerators across varied nursing duties. Participants noted a substantial reduction in task initiation time and overall completion cycles. Efficient data retrieval algorithms replaced traditional, time-consuming manual searches. By handling these low-value, repetitive tasks, AI provided notable psychological relief, reducing the subjective intensity and time pressure of administrative duties. As nurse N24 noted: “The main advantage is that it’s extremely efficient. You just send it your key images, data, and requirements, and it generates the output for you. It’s much more efficient than searching online yourself and analyzing or comparing things one by one; it’s very fast and convenient. It has reduced the amount of time I spend” (see Multimedia Appendix 5 for the full dataset).

###### Subtheme 3: Strengthening Team Synergy

Beyond individual use, embedded clinical AI systems (such as intelligent shift scheduling software and task distribution platforms) functioned as collaborative catalysts. These tools facilitated transparent role assignments and optimized team task distribution. In scenarios requiring collective decision-making, generative AI tools enabled the rapid integration of multidisciplinary information. Automated scheduling systems enhanced team synchronization and facilitated perceived collective outputs. As nurse N3 stated: “During group discussions, we can use AI to organize the questions we need to answer and better establish our future direction” (see Multimedia Appendix 5 for the full dataset).

### Theme 2: Clinical Cognitive Empowerment

This theme is conceptualized as the intellectual scaffolding provided by AI, which dynamically bridges individual knowledge gaps, reinforces clinical decision-making logic, and safeguards patient care in high-stakes clinical environments. In these complex settings, AI transcended its role as a mere physical tool to become an indispensable cognitive partner through instant knowledge replenishment, thinking logic scaffolding, and clinical safety safeguarding.

#### Subtheme 4: Instant Knowledge Replenishment

Nurses perceived generative AI tools and integrated clinical decision support systems as an on-demand knowledge repository that was particularly crucial during emergencies or unfamiliar cases. The systems provided rapid access to specialized clinical knowledge, complex pharmacological data (eg, drug interactions), and evidence-based guidelines. This instant access effectively masked experience deficits among junior nurses. As nurse N6 noted: “I use AI to organize and recall anatomical positions and functions; after sorting it out, I have a general idea of the clinical purpose and direction” (see Multimedia Appendix 5 for the full dataset).

#### Subtheme 5: Thinking Logic Scaffold

When confronting cognitive bottlenecks, such as complex case analyses or practice-based academic reflection, generative AI tools served as a cognitive coach. They provided structural frameworks, established logical connections between disparate symptoms, and assisted in structuring care plans. Participants emphasized that AI stimulated secondary reflection and expanded thinking dimensions. As nurse N8 described: “When I'm stuck on how to approach something, I might need AI to point the way. For example, when writing a report for the first time, I needed AI to give me a logical pivot. It also told me how to structure the logic of the text” (see Multimedia Appendix 5 for the full dataset).

#### Subtheme 6: Clinical Safety Safeguarding

Embedded clinical AI systems (eg, early warning scoring models and closed-loop smart infusion pumps) acted as vigilant partners in direct patient care. By monitoring microphysiological trends, predictive models offered early warnings for clinical deterioration, effectively reducing continuous vigilance fatigue. Automated assistance in high-risk procedures was perceived to enhance clinical safety monitoring and foster technological trust. As nurse N23 noted: “The intelligent early warning system mainly monitors patients’ vital signs... If any data is abnormal, it immediately alerts us so we can respond quickly” (see Multimedia Appendix 5 for the full dataset).

### Theme 3: Professional Capital Appreciation

Professional capital appreciation refers to the long-term enrichment of nurses’ psychological well-being, digital competencies, and occupational value, which fosters professional self-efficacy and enables a recentering on humanistic patient care. The use of AI generated these secondary benefits for nurses’ career trajectories through emotional energy replenishment, cross-boundary skill expansion, and core value regression.

#### Subtheme 7: Emotional Energy Replenishment

Delegating routine tasks to both generative AI tools and embedded clinical AI systems generated significant positive emotional energy. Nurses reported a heightened sense of control over their work pace and a reduction in digital anxiety. The reliable, on-demand nature of AI assistance served as a psychological buffer during overwhelming workloads. As nurse N4 shared: “Even though it’s complicated, we still have AI, right ?... It boosts my confidence in my work. With that confidence, I’m less likely to be bothered by difficulties” (see Multimedia Appendix 5 for the full dataset).

#### Subtheme 8: Cross-Boundary Skill Expansion

Generative AI tools functioned as accessible tutors that lowered thresholds for acquiring new digital competencies. Nurses used AI to bridge knowledge gaps, learn nursing research methodologies (eg, SPSS Statistics [Version 20.0; IBM Corp] operations and statistical workflows), and rapidly acquire digital content creation skills for patient education (eg, short video scripts). As nurse N33 shared: “It forced me to learn data analysis. Before, I just followed orders; now, I interpret AI-generated reports and analyze chronic disease trends in my district... It transformed me from an executor into a manager” (see Multimedia Appendix 5 for the full dataset).

#### Subtheme 9: Core Value Regression

A value-driven expectation emerged from the data. By absorbing trivial administrative and physical tasks, AI implicitly redirected nurses’ finite attention back to the essence of nursing: direct patient care and complex emotional labor. Participants expressed future aspirations for advanced AI—such as intelligent ward-roaming robots or automated health education loops—to fully eradicate manual drudgery, thereby allowing them to reclaim their professional identity as empathetic caregivers. As nurse N1 stated: “It can take over more mechanical tasks, such as documentation or monitoring, allowing nurses to focus more on patient psychological care and complex clinical management” (see Multimedia Appendix 5 for the full dataset).

### Domain 2: AI as a Job Demand (the Drain Path)

Viewed through the JD-R theoretical lens, the deployment of AI was not entirely benign. Instead, it introduced novel and complex job demands that triggered a potential health impairment process. Participants highlighted a perceived drain path, where the superficial alleviation of physical tasks was counterbalanced by subjective psychological, cognitive, and systemic burdens.

#### Theme 4: Cognitive Impediment

While AI promised cognitive relief, the unreliability and complex operational logic of current algorithms generated new dimensions of mental load, transforming nurses from care providers into algorithmic supervisors.

##### Subtheme 10: Verification Overload

A pervasive narrative was the persistent threat of algorithmic unreliability, which manifested distinct cognitive burdens across AI classes. For generative AI tools, nurses faced the threat of AI hallucinations (fabricated medical citations or inaccurate pharmacological data); for embedded clinical AI systems, nurses struggled with hypersensitive algorithms that caused frequent false alarms. Both mandated compulsory cross-verification, increasing cognitive fatigue and offsetting initial efficiency gains. As nurse N1 noted, “In individual diagnosis and treatment-related aspects, the results can vary significantly... We still need to cross-check with clinical experience or other case studies” (see Multimedia Appendix 5 for the full dataset).

##### Subtheme 11: Interaction Fatigue

The human-machine interaction associated with generative AI systems constituted a notable hidden demand. Participants described the high cognitive cost of prompt engineering, continuously adjusting, refining, and debugging instructions to align AI outputs with complex clinical realities or institutional frameworks. This cycle of trial and error interrupted workflows. As nurse N5 illustrated: “Initially, when giving instructions to AI, it often didn’t understand my intent, requiring constant refinement. Ambiguities inevitably arise, necessitating repeated questioning—this creates early-stage burdens”(see Multimedia Appendix 5 for the full dataset).

##### Subtheme 12: Mental Dependency

Long-term exposure to both generative AI tools and embedded clinical AI systems generated anxiety regarding professional deskilling. Nurses expressed deep concerns over cognitive blunting, fearing that outsourcing clinical reasoning, calculations, and diagnostic structuring to algorithms would atrophy their independent critical thinking and intuitive clinical judgment. As nurse N3 noted: “I unconsciously default to AI for task completion, bypassing independent thinking—this gradual reliance dulls cognitive engagement” (see Multimedia Appendix 5 for the full dataset).

### Theme 5: Relational Attrition

The insertion of algorithms into the health care ecosystem disrupted traditional human dynamics, generating novel interpersonal conflicts and threatening the empathetic core of nursing practice.

#### Subtheme 13: Interpretive Labor

The democratization of consumer-accessible generative AI tools meant that patients and families frequently used these systems for self-diagnosis. Frontline nurses were forced to absorb substantial amounts of new interpretive labor—deconstructing AI-generated misinformation, managing patient anxiety, and rebuilding trust. As nurse N30 stated: “Patients and families create challenges by consulting unreliable online sources... This undermines our health education efforts” (see Multimedia Appendix 5 for the full dataset).

#### Subtheme 14: Care Dehumanization

Participants harbored deep apprehensions regarding technology-mediated alienation caused by standardized algorithmic care pathways and automated communication scripts. Nurses feared a loss of humanistic care, perceiving algorithmic reliance as an emotional barrier that reduced holistic nursing to rigid, machine-dictated transactions. As nurse N12 noted, “Compared to humans, it’s deficient in humanistic care—a cornerstone of our profession” (see Multimedia Appendix 5 for the full dataset).

#### Subtheme 15: Professional Alienation

Embedded clinical AI systems (eg, performance-tracking algorithms and automated shift scheduling) and rapid technology iterations fostered professional insecurity. Senior staff facing the digital divide experienced anxiety regarding displacement and feeling managed by algorithms, which eroded their clinical agency. As nurse N23 described: “My greatest fear is AI wholly supplanting nursing roles, eliminating job opportunities” (see Multimedia Appendix 5 for the full dataset).

### Theme 6: Digital Involution

In the highly competitive context of the modern health care system, digital involution is conceptualized as a sociotechnical paradox wherein technology-driven efficiency artificially inflates performance expectations, locking nurses into a cycle of hyper-competition, perfectionism, and invisible labor without proportional gains in well-being. Specifically, the integration of AI catalyzed unintended structural pressures across 3 interrelated dimensions: performance inflation, competitive perfectionism, and workload expansion.

#### Subtheme 16: Performance Inflation

The accessibility and speed of generative AI tools lowered the threshold for complex administrative and quality-improvement deliverables (eg, report synthesis or presentation design), which paradoxically triggered performance inflation. Management implicitly raised baseline standards, demanding perfect deliverables and forcing nurses into a cycle of continuous overperformance. As nurse N33 illustrated: “Management increasingly judges quality by numbers rather than substance. Quantity gradually overshadows quality focus, and performance benchmarks keep escalating.” (see Multimedia Appendix 5 for the full dataset).

#### Subtheme 17: Competitive Perfectionism

The accessibility of AI-generated content fostered an environment of intensified peer comparison. Nurses felt compelled to produce flawlessly polished materials to maintain their professional standing, leading to a state of competitive perfectionism. This subtheme highlights how AI-driven high standards became the new minimum requirement, forcing individuals to invest excessive cognitive effort into refining AI outputs to avoid being outperformed by colleagues. As nurse N23 noted: “I have to spend time after work every day learning related knowledge, and I feel a lot of pressure. Before, when we wrote health education materials, ‘good enough’ was fine. But now with AI, the requirements keep getting higher, the quality keeps improving — it’s very competitive” (see Multimedia Appendix 5 for the full dataset).

#### Subtheme 18: Workload Expansion

Contrary to labor-saving promises, findings reveal a paradoxical expansion of invisible digital labor across both technology classes. This includes troubleshooting embedded system errors, performing redundant dual-entry processes between legacy systems and AI platforms, and debugging generative AI outputs. As nurse N7 noted: “Nurses haven’t had responsibilities reduced by AI; instead, we’ve gained the added duty of monitoring AI for malfunctions” (see Multimedia Appendix 5 for the full dataset).

### Domain 3: Perceived Buffering Conditions for Navigating the Dual-Edged Sword Effect of AI

Within the JD-R model, the transition of AI from a potential job demand back to an empowering job resource is heavily contingent on specific buffering conditions. The analysis revealed that nurses actively and passively used various moderating mechanisms to navigate the double-edged nature of AI. These mechanisms, which mitigate the health impairment process (ie, the strain path where high job demands deplete emotional and cognitive energy, leading to burnout) and enhance the motivational process (ie, the gain path where abundant job resources foster work engagement and self-efficacy), are conceptualized into 3 core themes: proactive coping strategies, professional boundary demarcation, and organizational support architecture.

#### Theme 7: Proactive Coping Strategies

Nurses did not remain passive recipients of technological outputs; instead, they engaged in highly proactive behavioral crafting to harness AI’s use while neutralizing its risks.

##### Subtheme 19: Critical Verification Execution

To combat generative AI hallucinations and predictive model false alarms, nurses developed rigorous cross-verification habits. Rather than blindly trusting algorithmic outputs, participants used multisource cross-verification strategies, treating AI-generated content purely as a preliminary draft rather than definitive evidence. This active cognitive filtering served as a crucial buffer, preventing machine errors from translating into clinical incidents. As nurse N6 illustrated: “If findings contradict my foundational knowledge, I reevaluate and reassess AI’s conclusions” (see Multimedia Appendix 5 for the full dataset).

##### Subtheme 20: Strategic Interaction Mastery

Nurses ameliorated interaction friction, specifically when using generative AI tools, by mastering prompt engineering, strategic data feeding (providing authentic clinical contexts), and task-technology calibration (delegating low-risk administrative drafting to AI while reserving complex clinical decisions for traditional human pathways). As nurse N15 shared: “Command refinement is key—iterative tweaks align outputs with needs” (see Multimedia Appendix 5 for the full dataset).

### Theme 8: Professional Boundary Demarcation

A robust psychological and professional defense mechanism emerged as nurses deliberately fortified their occupational identity against technological encroachment.

#### Subtheme 21: Role Positioning Clarity

Participants conceptualized both generative AI tools and embedded clinical AI systems strictly as high-capacity subordinate assistants or information aggregators, vehemently rejecting AI as an autonomous decision-maker. Recognizing AI’s lack of genuine empathy insulated nurses from replacement anxiety. As nurse N26 noted: “A textual assistant. For reference and support—nothing more” (see Multimedia Appendix 5 for the full dataset).

#### Subtheme 22: Clinical Agency Retention

Nurses preserved clinical autonomy by insisting on the irreplaceable nature of physical touch and empathetic care. In clinical decision-making, they adopted a “think first, consult second” paradigm, ensuring human reasoning framed the clinical strategy. As nurse N6 stated: “*I adhere to my own logic—AI won’t override my reasoning. Critical thinking is non-negotiable. With independent thought, AI becomes a tool, not a puppeteer* ”(see Multimedia Appendix 5 for the full dataset).

### Theme 9: Organizational Support Architecture

The organizational environment served as a macroscopic macroregulator. The presence or absence of institutional backing dictated whether AI integration felt like an empowering resource or a competitive burden.

#### Subtheme 23: Systemic Resource Provision

Participants expressed a need for hospitals to transcend fragmented usage by deploying unified, institutionally sanctioned, vertically integrated medical AI platforms (eg, specialized medical LLMs trained on validated clinical guidelines). Centrally provided platforms lower cognitive thresholds and alleviate security concerns. As nurse N3 noted: “Hospitals could consolidate databases... Institution-led AI platforms would streamline retrieval” (see Multimedia Appendix 5 for the full dataset).

#### Subtheme 24: Capacity Building Support

Nurses emphasized the need for structured organizational training focusing on underlying algorithmic logic (for embedded clinical AI systems) and prompt structuring and error avoidance (for generative AI tools), backed by clear institutional policies and ethical boundaries. As nurse N3 described: “If hospitals adopt AI, they should develop structured teaching programs” (see Multimedia Appendix 5 for the full dataset).

## Discussion

### Summary of Principal Findings

Guided by the JD-R model, this qualitative study provides a nuanced qualitative empirical understanding of the double-edged sword effect of AI integration among frontline clinical nurses. Our findings demystify AI from being a unilaterally empowering artifact, revealing it instead as a highly dynamic agent that concurrently activates distinct gain and drain pathways. On the gain path, AI functions as a robust job resource that initiates a motivational process, characterized by a substantial workflow efficiency leap, critical clinical cognitive empowerment, and the subsequent appreciation of professional capital. Conversely, the drain path exposes the hidden sociotechnical costs where AI operates as a novel, multifaceted job demand. These demands manifest as cognitive friction, relational strain that threatens traditional empathetic care, and a pervasive systemic involution driven by technology-induced efficiency inflation. Crucially, our qualitative findings suggest that this technological paradox is not perceived as deterministic. Rather than demonstrating tested causal moderation, our data illustrate that the lived interplay between AI-driven resources and demands is contextualized by key perceived buffering conditions: nurses’ proactive coping strategies, their rigorous professional boundary demarcation to preserve clinical agency, and the provision of a cohesive organizational support architecture.

### The Double-Edged Sword Effect: Deconstructing the Gain and Drain Pathways

#### The Gain Path

Consistent with the motivational pathway of the JD-R model, our findings validate that AI serves as a potent perceived job resource, initiating empowerment across 3 progressive dimensions: operational workflow, clinical cognition, and professional capital. First, regarding operational execution, our findings corroborate historical health information technology literature that emphasizes the physical and temporal benefits of automation [25]. We demonstrate a substantial workflow efficiency leap, where disaggregated technology classes yield distinct operational resources: generative AI tools streamline documentation automation and literature retrieval, whereas embedded clinical AI systems enhance shift scheduling and team synergy. These observations converge with recent qualitative scholarship, highlighting AI’s capacity to alleviate administrative burdens and optimize workflow management [6,21,26]. Beyond routine automation, AI provides critical clinical cognitive empowerment. Specifically, embedded clinical AI systems (eg, early warning scores) act as safety sentinels, whereas generative AI tools function as on-demand cognitive tutors that bridge knowledge gaps and scaffold clinical reasoning. This positions AI as an active adjunct to human cognition [27-29], directly boosting nurses’ self-efficacy [30,31] and rendering complex tasks manageable [32]. Ultimately, this operational and cognitive synergy fosters professional capital appreciation. By delegating mundane tasks to algorithms, nurses restore emotional resources, acquire cross-boundary digital skills, and experience a “core value regression”—redirecting finite attentional capacity back to the irreplaceable, humanistic core of patient care.

#### The Drain Path

Despite the optimistic narrative surrounding AI empowerment, our study uncovers a perceived health impairment process driven by novel digital demands. Consistent with Chuang et al [33], who demonstrated the dual impact of AI on work and life well-being under the JD-R model, our finding reinforces the model’s suitability for unpacking the paradoxical effects of AI in clinical nursing contexts. A critical finding in this study is the manifestation of an AI productivity discrepancy rooted in cognitive impediment, which manifests through distinct technological mechanisms. While AI theoretically accelerates task completion, this temporal advantage is frequently neutralized by hidden cognitive loads. For generative AI tools, cognitive load stems from the threat of algorithmic hallucinations and omissions, forcing nurses into tedious prompt engineering and multisource verification; for embedded clinical AI systems, cognitive strain is driven by hypersensitive false alerts and alert fatigue [34]. As our data suggests, cognitive workload has not disappeared; it has merely shifted from primary content creation to secondary content verification. This verification pressure is further compounded by the psychological risk of automation bias (uncritical overreliance on AI outputs) versus the cognitive friction of overriding false alerts.

Furthermore, an important finding is that within the highly competitive environment of contemporary health care, the widespread accessibility of generative AI tools has inadvertently driven performance inflation. As AI lowers the technical barriers for producing polished administrative or quality-improvement work, nursing management implicitly raises baseline expectations. While prior literature often conceptualizes technological stress as individual operational difficulty, our findings uncover a novel sociotechnical phenomenon—digital involution—wherein frontline nurses are locked into a cycle of competitive perfectionism and invisible workload expansion (eg, dual-entry validation and prompt debugging). When combined with the interpretive labor required to bridge the gap between AI misinformation and patient understanding amid ambiguous legal responsibility frameworks, these demands constitute a substantial source of technostress. If left unaddressed, this invisible surge in task intensity, coupled with professional alienation and the fear of occupational displacement, threatens the long-term sustainability of the nursing workforce. These findings suggest that without organizational interventions to decouple AI efficiency from ever-increasing performance quotas, the double-edged sword of AI may inadvertently accelerate burnout rather than alleviate it.

### Perceived Buffering Conditions: Navigating Demands and Resources

Another significant finding is that our qualitative findings illustrate how nurses perceive AI as shifting between a resource and a demand depending on contextual buffering conditions at individual, professional, and organizational levels. At the micro level, nurses exercise agency through proactive coping and strategic interaction proficiency [34]—tailoring prompt engineering for generative LLMs while maintaining vigilant verification for embedded clinical alerts. By selectively delegating routine tasks to algorithms while retaining high-stakes clinical judgments, practitioners optimize operational gains while mitigating cognitive risks. This resilience is anchored in professional boundary demarcation, where AI is positioned strictly as an auxiliary tool rather than an autonomous decision-maker. Such role clarity, combined with an emphasis on humanistic care, serves as a cognitive buffer against occupational replacement anxiety and technological alienation.

However, individual resilience alone is insufficient to sustain this balance. Organizational support architectures serve as the critical fulcrum. To prevent a cycle in which efficiency gains from technology inadvertently trigger escalating performance quotas, health care institutions must provide systemic resources. These include institutionally sanctioned, vertically integrated medical AI models (to eliminate the privacy and hallucination risks of generic public chatbots) and structured capacity-building programs focused on algorithmic logic, prompt calibration, and error avoidance. Ultimately, the synergy between individual professional agency and institutional scaffolding helps explain whether AI is perceived as an occupational hazard or a sustainable clinical resource.

### Implications for Nursing Management and Practice

The findings of this study offer critical and actionable insights for health care administrators seeking to sustainably integrate AI into clinical nursing practice without exacerbating burnout.

First, to mitigate the cognitive impediments and legal anxiety associated with algorithmic hallucinations and false alerts, nursing administrators should actively collaborate with interprofessional hospital leadership to co-design and implement nursing-specific AI governance guidelines within the broader organizational framework [35]. For instance, nurse leaders can establish clear operational protocols that delineate permissible AI boundaries—such as defining mandatory “human-in-the-loop” verification steps for AI-predictive risk alerts and establishing explicit guidelines that mandate clinical nurse sign-off before AI-generated documentation or care plans are finalized in patient records.

Second, health care institutions must establish a comprehensive, multidimensional AI life cycle governance framework encompassing people, processes, technology, and operations [36]. Rather than assuming that adopting institutionally sanctioned, vertically integrated medical AI models automatically eliminates privacy or reliability risks, hospital management must recognize that technological infrastructure requires robust operational oversight. Centrally funded and domain-specific clinical AI tools (eg, algorithms trained on validated guidelines and secure electronic health record databases) should be deployed alongside strict operational protocols: enforcing mandatory data deidentification, embedding clear digital audit trails, defining legal liability boundaries, and establishing explicit escalation procedures when algorithmic outputs conflict with human clinical judgment. Crucially, organizational policies must safeguard against automation bias and model drift by institutionalizing mandatory human oversight and conducting routine audits of joint human–AI system performance under both optimal and misleading AI outputs.

Third, the paradigm of capacity building support must evolve. Nursing education and continuous professional development programs should move beyond rudimentary software tutorials to cultivate comprehensive AI literacy. This involves equipping nurses with advanced prompt engineering techniques for generative AI tools, algorithmic awareness for embedded clinical AI systems, and standardized critical verification protocols.

Finally, to dismantle the escalating cycle of performance demands driven by technology, management must fundamentally recalibrate performance evaluation metrics. It is imperative that leadership ensures the temporal resources liberated by AI are channeled into a core value regression. Specifically, time saved should be redirected back to direct patient care and empathetic engagement rather than being absorbed by artificially inflated administrative workloads or escalating performance targets.

### Strengths, Limitations, and Future Research

A key strength of this study lies in its robust theoretical anchorage within the JD-R model, providing theoretically grounded qualitative insights into the lived experiences of frontline nurses navigating AI. However, several limitations warrant acknowledgment. First, as a cross-sectional qualitative content analysis, this study captures participants’ perceived experiences and subjective reflections; it cannot establish statistical causality, measure objective clinical performance/safety outcomes, or formally test quantitative mediation or moderation pathways. Second, because data were collected exclusively within mainland China, specific culturally embedded psychosocial dynamics may lack statistical generalizability and could manifest differently across diverse global health care ecosystems. Third, the reliance on digital recruitment strategies and voluntary participation may introduce self-selection bias toward hyper-users or tech-savvy individuals. Consequently, future research should prioritize the development and validation of psychometric instruments to quantitatively test these newly identified AI-driven JD-R pathways within larger, representative populations. Furthermore, longitudinal designs and cross-cultural comparative studies are strongly recommended to continuously track the enduring impacts of algorithmic integration on occupational well-being, evaluate joint human–AI system performance under both optimal and misleading AI conditions, and elucidate how varying organizational support architectures globally buffer these complex technological demands.

### Conclusions

This qualitative study provides a nuanced theoretical unpacking of the double-edged sword effect of AI in clinical nursing through the lens of the JD-R model. While AI acts as an empowering job resource by facilitating perceived efficiency gains and clinical cognitive empowerment, it simultaneously introduces notable digital job demands. These demands manifest primarily as cognitive impediments stemming from verification overload, relational attrition challenging the empathetic core of nursing, and the psychological exhaustion induced by digital involution. Crucially, our findings illustrate that the trajectory of AI integration is not technologically deterministic. Instead, the lived interplay between AI-driven resources and demands is contextualized by key perceived buffering conditions: nurses’ proactive coping strategies, rigorous professional boundary demarcation, and the provision of a robust organizational support architecture. To prevent AI from devolving into an occupational hazard, health care administrators must transcend the mere deployment of algorithms. By establishing comprehensive life cycle AI governance, providing advanced AI literacy training, and safeguarding the irreplaceable humanistic essence of the profession, organizations can sustainably harness AI to empower the nursing workforce and elevate patient care in the digital era.

## Supplementary material

10.2196/100916 Multimedia Appendix 1 The Job Demands‐Resources model.

10.2196/100916 Multimedia Appendix 2 Interview outline based on the Job Demands-Resources model.

10.2196/100916 Multimedia Appendix 3 Participant characteristics (n=34).

10.2196/100916 Multimedia Appendix 4 Participant AI exposure matrix.

10.2196/100916 Multimedia Appendix 5 Thematic coding tree based on the Job Demands-Resources model.

## Abbreviations

DQCA: directed qualitative content analysis
JD-R: Job Demands-Resources
LLM: large language model
SRQR: Standards for Reporting Qualitative Research
