# Supplementary material for: The Double-Edged Sword Effect of AI Application Among Clinical Nurses Based on the Job Demands-Resources Model: Qualitative Study
Source: JMIR Nurs. 2026 Oct 2;9:e100916. doi: 10.2196/100916 (PMC13632912; doi:10.2196/100916)
Supplement: Supplementary file 1 [file nursing-v9-e100916-s001.doc]

**Multimedia Appendix 1. The job demands‐resources model**
