# Supplementary material for: The Double-Edged Sword Effect of AI Application Among Clinical Nurses Based on the Job Demands-Resources Model: Qualitative Study
Source: JMIR Nurs. 2026 Oct 2;9:e100916. doi: 10.2196/100916 (PMC13632912; doi:10.2196/100916)
Supplement: Supplementary file 2 [file nursing-v9-e100916-s002.doc]

**Multimedia Appendix 2. Interview outline based on the JD-R model**

| Part 1: Background Information and Initial Introduction | 1. Please briefly describe your clinical department, years of work experience, and primary job responsibilities. 2. In your current work, which artificial intelligence (AI)-related tools or systems have you been exposed to or used? (Examples: deepseek, Wenxin Yiyan, intelligent early warning systems, infusion pumps, voice-to-text medical record entry, risk prediction models, scheduling systems, etc.) 3. Please describe a recent, specific instance in which you used an AI tool to complete a particular work task. |
| --- | --- |
| Part 2: Job Demands Experiences Based on the JD-R Model | 4. Has learning, adapting to, or operating this AI system imposed additional learning pressure or cognitive burden on you? Could you provide an example? 5. Has using AI made certain work processes more complex, increased the number of steps, or interrupted your original work rhythm? 6. Has the introduction of AI affected your communication with patients, their families, or colleagues? Do you need to spend more effort explaining or coordinating AI‑related matters? 7. Has the data or evaluation results generated by AI brought new performance pressure to you? 8. Have you ever felt anxious or frustrated due to AI system malfunctions, false alarms, or inaccuracies, or have you needed to invest extra time in troubleshooting or verification? |
| Part 3: Job Resources Experiences Based on the JD-R Model | 9. In what ways has AI acted like an assistant, directly saving you time, physical effort, or simplifying tedious tasks? 10. To what extent have the data, alerts, or predictions provided by AI helped you make more timely and accurate clinical judgments or decisions? 11. Has AI increased your professional knowledge or skills in any way? How has it influenced your learning? 12. Has AI given you a stronger sense of control over your work (e.g., more reasonable time management), or do you feel constrained or dominated by the system? 13. Has AI improved information sharing and collaboration among your team? Alternatively, has it provided you with unprecedented objective feedback regarding your work patterns? |
| Part 4: Balancing the Two-Edged Sword, Outcomes, and Organisational Support | 14. Overall, does using AI make you feel more energetic and engaged at work, or more emotionally exhausted and detached? Or both? 15. Have you ever experienced a moment when the “help” and “trouble” of AI coexisted? How did you weigh and handle the situation at that time? 16. In your opinion, what kinds of personal experience, training, or team climate can maximise the benefits of AI while minimising its downsides? 17. What specific support (e.g., training, process optimisation, psychological support) would you hope the hospital or department management to provide to help you better collaborate with AI and mitigate its negative impacts? |
| Part 5: Summary and Future Perspectives | 18. If you were to use a metaphor to describe AI’s role in your work (for example: “Is it like an indefatigable apprentice, or a mechanical colleague that occasionally makes trouble?”), what metaphor would you choose? Why? 19. Regarding deeper future applications of AI in nursing, what is your greatest expectation? What is your greatest concern? 20. Is there anything we have not yet discussed that you believe is crucial to understanding nurses’ experiences with using AI? |
