# Supplementary material for: The Double-Edged Sword Effect of AI Application Among Clinical Nurses Based on the Job Demands-Resources Model: Qualitative Study
Source: JMIR Nurs. 2026 Oct 2;9:e100916. doi: 10.2196/100916 (PMC13632912; doi:10.2196/100916)
Supplement: Supplementary file 3 [file nursing-v9-e100916-s003.doc]

**Multimedia Appendix 3. Participant characteristics (n = 34)**

| **ID** | **Age (years)** | **Gender** | **Education** | **Title** | **Departments** | **Nursing experience (years)** | **Hospital tier** |
| --- | --- | --- | --- | --- | --- | --- | --- |
| N1 | 24 | Female | Undergraduate | Junior | Surgery | 2 | Tertiary |
| N2 | 27 | Female | Junior College or below | Junior | Internal medicine | 6 | Tertiary |
| N3 | 29 | Male | Undergraduate | Intermediate | Emergency | 7 | Secondary |
| N4 | 26 | Male | Undergraduate | Junior | Surgery | 4 | Tertiary |
| N5 | 31 | Male | Undergraduate | Intermediate | ICU | 10 | Tertiary |
| N6 | 38 | Male | Junior College or below | Intermediate | Surgery | 17 | Secondary |
| N7 | 43 | Female | Postgraduate | Senior | Surgery | 18 | Tertiary |
| N8 | 25 | Male | Junior College or below | Junior | Internal medicine | 4 | Tertiary |
| N9 | 30 | Male | Undergraduate | Intermediate | Surgery | 8 | Tertiary |
| N10 | 34 | Male | Postgraduate | Intermediate | Internal medicine | 9 | Tertiary |
| N11 | 23 | Female | Undergraduate | Junior | Surgery | 1 | Tertiary |
| N12 | 27 | Male | Undergraduate | Junior | Emergency | 5 | Secondary |
| N13 | 32 | Male | Undergraduate | Intermediate | Internal medicine | 11 | Tertiary |
| N14 | 29 | Male | Undergraduate | Intermediate | Surgery | 7 | Tertiary |
| N15 | 28 | Male | Postgraduate | Intermediate | ICU | 3 | Tertiary |
| N16 | 36 | Female | Undergraduate | Intermediate | Internal medicine | 15 | Secondary |
| N17 | 23 | Female | Junior College or below | Junior | Internal medicine | 2 | Tertiary |
| N18 | 25 | Female | Undergraduate | Junior | Emergency | 4 | Tertiary |
| N19 | 31 | Female | Undergraduate | Intermediate | Surgery | 9 | Tertiary |
| N20 | 29 | Female | Undergraduate | Intermediate | Internal medicine | 7 | Tertiary |
| N21 | 34 | Female | Undergraduate | Intermediate | Surgery | 13 | Tertiary |
| N22 | 24 | Female | Undergraduate | Junior | Surgery | 2 | Secondary |
| N23 | 35 | Female | Postgraduate | Intermediate | ICU | 10 | Tertiary |
| N24 | 37 | Female | Postgraduate | Senior | Internal medicine | 14 | Tertiary |
| N25 | 26 | Female | Undergraduate | Junior | Internal medicine | 4 | Tertiary |
| N26 | 30 | Male | Undergraduate | Intermediate | Emergency | 8 | Tertiary |
| N27 | 33 | Male | Postgraduate | Intermediate | Surgery | 8 | Tertiary |
| N28 | 42 | Female | Undergraduate | Senior | Internal medicine | 22 | Secondary |
| N29 | 27 | Female | Undergraduate | Intermediate | ICU | 6 | Tertiary |
| N30 | 31 | Female | Undergraduate | Intermediate | Surgery | 9 | Tertiary |
| N31 | 25 | Female | Undergraduate | Junior | Surgery | 4 | Tertiary |
| N32 | 39 | Female | Undergraduate | Intermediate | Internal medicine | 17 | Community |
| N33 | 40 | Female | Postgraduate | Senior | Internal medicine | 19 | Tertiary |
| N34 | 28 | Female | Undergraduate | Intermediate | Surgery | 6 | Secondary |
