# Supplementary material for: The Double-Edged Sword Effect of AI Application Among Clinical Nurses Based on the Job Demands-Resources Model: Qualitative Study
Source: JMIR Nurs. 2026 Oct 2;9:e100916. doi: 10.2196/100916 (PMC13632912; doi:10.2196/100916)
Supplement: Supplementary file 4 [file nursing-v9-e100916-s004.doc]

**Multimedia Appendix 4. Participant AI exposure matrix (n = 34)**

| **ID** | **Specific AI Tool/System Used** | **Institutional Approval Status** | **Primary Task Domain** | **Frequency of Use** | **Data Entered & Privacy Protocol** | **Training Received** | **Degree of Workflow Integration** |
| --- | --- | --- | --- | --- | --- | --- | --- |
| N1 | (1) Doubao  (2) DeepSeek  (3) Baidu AI | (1-3) Personally Accessed | (1) Document drafting  (2) Information retrieval  (3) PPT making | (1-2) Daily  (3) Sporadic | (1-3) Manual de-identified text | (1-3) Self-taught | (1) Low  (2) Moderate  (3) Low |
| N2 | (1) DeepSeek  (2) Doubao  (3) Kimi  (4) ChatGPT  (5) Bard | (1-5) Personally Accessed | (1) Information retrieval  (2) Mind mapping  (3) PPT making  (4) Information retrieval  (5) Information retrieval | (1-2) Regular  (3-5) Occasional | (1-5) Manual de-identified text | (1-5) Self-taught | (1) Moderate  (2-5) Low |
| N3 | (1) DeepSeek  (2) Doubao  (3) WPS AI  (4) Baidu AI  (5) Early Warning Score System | (1-4) Personally Accessed  (5) Hospital-Integrated | (1) Literature reading & summarization  (2) Information retrieval  (3) PPT making  (4) Image editing  (5) Patient deterioration risk monitoring | (1-4) Occasional  (5) Daily | (1-4) Manual de-identified text  (5) Real-time EHR vitals | (1-4) Self-taught  (5) Hospital Onboarding | (1) Low  (2) Moderate  (3) Moderate  (4) Low  (5) High |
| N4 | (1) DeepSeek  (2) Automated Electronic Health Record Data Extraction | (1) Personally Accessed (2) Hospital-Integrated | (1) Statistical method learning & code generation, English translation & polishing  (2) Auto-extract patient data nursing | (1) Regular  (2) Regular | (1) Manual de-identified research data  (2) Real-time EHR data | (1) Self-taught (2) Hospital Onboarding | (1) Moderate  (2) High |
| N5 | (1) Wenxin Yiyan (2) Doubao  (3) DeepSeek  (4) Physiological Monitoring Algorithm | (1-3) Personally Accessed  (4) Hospital-Integrated | (1) Problem retrieval & idea supplement  (2) PPT making  (3) Mplus code debugging & modification  (4) Real-time vital sign trend analysis | (1-3) Occasional  (4) Daily | (1-3) Manual de-identified text  (4) Real-time EHR vitals | (1-3) Self-taught  (4) Hospital Onboarding | (1-3) Low  (4) High |
| N6 | (1) DeepSeek  (2) Mita AI  (3) Bard  (4) Risk Prediction System | (1-3) Personally Accessed  (4) Hospital-Integrated | (1) Initial problem exploration  (2) Literature retrieval  (3) Research methodology consultation, Interview guide drafting, Clinical knowledge retrieval  (4) Post-op venous thrombus embolism risk stratification | (1-3) Occasional  (4) Daily | (1-3) Manual de-identified text  (4) Real-time EHR data | (1-3) Self-taught  (4) Hospital Onboarding | (1-3) Low  (4) High |
| N7 | (1) Smart Infusion System (2) Risk Prediction System (3) Imaging Analysis System (4) DeepSeek (5) Clinical Decision Support System | (1-3, 5) Hospital-Integrated  (4) Personally Accessed | (1) Real-time infusion monitoring & alerting  (2) Venous thrombus embolism risk prediction  (3) Medical image interpretation  (4) Patient education material generation  (5) Automated care plan recommendation & nursing diagnosis support | (1-3, 5) Daily  (4) Occasional | (1-3, 5) Real-time EHR data  (4) Manual de-identified text | (1-3, 5) Hospital Onboarding  (4) Self-taught | (1-3, 5) High  (4) Low |
| N8 | (1) DeepSeek  (2) ChatGPT  (3) Baidu AI  (4) Bard  (5) Clinical Decision Support System | (1-4) Personally Accessed  (5) Hospital-Integrated | (1) Information retrieval  (2) Academic literature search  (3) logic structuring  (4) Software learning  (5) Diagnostic reminder & care plan suggestion | (1-4) Occasional  (5) Daily | (1-4) Manual de-identified text  (5) Real-time EHR data | (1-4) Self-taught  (5) Hospital Onboarding | (1-4) Low  (5) High |
| N9 | (1) DeepSeek  (2) Doubao  (3) Wenxin Yiyan (4) ChatGPT  (5) Bard  (6）Tongyi Qianwen  (7) Intelligent Shift Scheduling System | (1-6) Personally Accessed  (7) Hospital-Integrated | (1) PDF literature reading & summarization  (2) Literature retrieval  (3) Image generation  (4) Text polishing  (5) Reports writing  (6) Knowledge summarization for exam preparation, Team task division  (7) Automated scheduling | (1-6) Occasional  (7) Daily | (1-6) Manual de-identified text  (7) Staffing & patient acuity data | (1-6) Self-taught  (7) Hospital Onboarding | (1-6) Low  (7) High |
| N10 | (1) ChatGPT  (2) Doubao  (3) DeepSeek  (4) Clinical Decision Support System | (1-3) Personally Accessed  (4) Hospital-Integrated | (1) Activity outline/PPT framework generation  (2) Team information collection & data screening  (3) Information retrieval  (4) Nursing plan | (1-3) Occasional  (4) Daily | (1-3) Manual de-identified text  (4) Real-time EHR data | (1-3) Self-taught  (4) Hospital Onboarding | (1-3) Low  (4) High |
| N11 | (1) Doubao  (2) DeepSeek  (3) Automated Electronic Health Record Data Extraction | (1-2) Personally Accessed  (3) Hospital-Integrated | (1) Daily information Q&A  (2) Text polishing & enrichment, Drug pharmacology queries, Team task framework structuring  (3) Auto-generate shift handover reports | (1-2) Regular  (3) Daily | (1-2) Manual de-identified text (3) Real-time EHR data | (1-2) Self-taught  (3) Hospital Onboarding | (1-2) Low  (3) High |
| N12 | (1) DeepSeek  (2) Doubao  (3) ChatGPT  (4) Triage Decision Support System | (1-3) Personally Accessed  (4) Hospital-Integrated | (1) Information retrieval & data integration  (2) Expression optimization & polishing, Understanding complex theoretical knowledge, Image editing (photo retouching)  (3-4) Acuity-based triage nurisng guidance | (1-3) Occasional (4) Daily | (1-3) Manual de-identified text (4) Real-time patient data | (1-3) Self-taught \  (4) Hospital Onboarding | (1-3) Low  (4) High |
| N13 | (1) DeepSeek  (2) Doubao  (3)Wenxin Yiyan (4) Risk Prediction System | (1-3) Personally Accessed  (4) Hospital-Integrated | (1) Content generation  (2) Image generation & editing  (3) Photo-to-text conversion & table generation, Proposal review & modification  (4) Fall risk scoring & prevention alert | (1-3) Occasional (4) Daily | (1-3) Manual de-identified text (4) Real-time EHR mobility data | (1-3) Self-taught  (4) Hospital Onboarding | (1-3) Low  (4) High |
| N14 | (1) Wenxin Yiyan  (2) Kimi  (3) Quark Qianwen  (4) Intelligent Wound Assessment System | (1-3) Personally Accessed  (4) Hospital-Integrated | (1) Text polishing & optimization  (2) Summary/report drafting  (3) Statistical problem-solving  (4) AI-assisted wound photo analysis & staging | (1-3) Occasional (4) Regular | (1-3) Manual de-identified text (4) Uploaded wound images (de-identified) | (1-3) Self-taught  (4) Hospital Onboarding | (1-3) Low  (4) Moderate |
| N15 | (1) Doubao  (2) DeepSeek | (1-2) Personally Accessed | (1) Image search for PPT (2) Outline/framework generation, Information retrieval, Image text extraction & organization, Clinical case learning analysis | (1-2) Occasional | (1-2) Manual de-identified text | (1-2) Self-taught | (1-2) Moderate |
| N16 | (1) ChatGPT  (2) DeepSeek  (3) Doubao  (4) Risk Prediction System | (1-3) Personally Accessed  (4) Hospital-Integrated | (1) Text polishing  (2) Proposal draft generation & revision  (3) Knowledge supplementation & concept connection, Daily life tips (4) High-risk patient identification | (1-3) Occasional (4) Daily | (1-3) Manual de-identified text (4) Real-time EHR/lab data | (1-3) Self-taught  (4) Hospital Onboarding | (1-3) Low  (4) High |
| N17 | (1) Doubao  (2) DeepSeek  (3) Automated Electronic Health Record Data Extraction | (1-2) Personally Accessed  (3) Hospital-Integrated | (1) Science video/animation generation (2) Image generation, Video script generation, PPT making, Daily search  (3) Auto-extract quality indicators for reporting | (1-2) Regular  (3) Daily | (1-2) Manual de-identified text (3) Real-time EHR data | (1-2) Self-taught  (3) Hospital Onboarding | (1-2) Low  (3) High |
| N18 | (1) DeepSeek  (2) Doubao  (3) Kimi  (4) Smart Infusion System | (1-3) Personally Accessed  (4) Hospital-Integrated | (1) Text-based information search  (2) PPT/teaching courseware making  (3) Information summarization & categorization, Drug usage  (4) Real-time rate adjustment & occlusion alarm | (1-3) Occasional (4) Daily | (1-3) Manual de-identified text (4) Real-time infusion data | (1-3) Self-taught  (4) Hospital Onboarding | (1-3) Low  (4) High |
| N19 | (1) DeepSeek  (2) Doubao  (3) Smart Display System  (4) Risk Prediction System | (1-2) Personally Accessed  (3-4) Hospital-Integrated | (1) Current affairs analysis (2) Clinical question Q&A (pharmacology, chemotherapy, wound care) (3) Teaching/lecture display (video, PPT)  (4) Post-op venous thrombus embolism risk alert | (1-2) Occasional  (3-4) Daily | (1-2) Manual de-identified text (3) Hospital intranet data (4) Real-time EHR data | (1-2) Self-taught  (3-4) Hospital Onboarding | (1-2) Low  (3-4) High |
| N20 | (1) DeepSeek | (1) Personally Accessed | (1) PPT outline generation, Health education material writing, Article logic optimization | (1) Occasional | (1) Manual de-identified text | (1) Self-taught | (1) Low |
| N21 | (1) DeepSeek  (2) Doubao  (3) Smart Triage Guidance System | (1-2) Personally Accessed  (3) Hospital-Integrated | (1) PPT making  (2) Publicity material drafting  (3) Patient triage guidance | (1-2) Occasional  (3) Daily | (1-2) Manual de-identified text  (3) Real-time patient data | (1-2) Self-taught  (3) Hospital Onboarding | (1-2) Low  (3) High |
| N22 | (1) ChatGPT  (2) DeepSeek  (3) Doubao  (4) Kimi  (5) Smart Follow-up System | (1-4) Personally Accessed  (5) Hospital-Integrated | (1) Statistical software guidance (SPSS, Mplus code)  (2) Proposal writing & polishing  (3) Speech/emcee script writing  (4) Image generation for PPT, Paper writing & language polishing, Clinical knowledge retrieval, Emotional venting  (5) Automated post-discharge follow-up & health monitoring | (1-4) Regular  (5) Daily | (1-4) Manual de-identified text  (5) Real-time EHR data | (1-4) Self-taught  (5)  Hospital Onboarding | (1-4) Low/Moderate  (5)  High |
| N23 | (1) Early Warning System  (2) Intelligent Scheduling System  (3) Diagnostic Assistance Systeme System | (1-3) Hospital-Integrated | (1) Real-time vital sign monitoring & alerting  (2) Automated shift scheduling  (3) Symptom-based differential diagnosis support | (1-3) Daily | (1-3) Real-time EHR data | (1-3) Hospital Onboarding | (1-3) High |
| N24 | (1) DeepSeek  (2) Clinical Decision Support System | (1) Personally Accessed (2) Hospital-Integrated | (1) Summary writing, Material drafting, Information retrieval  (2) Chronic disease management | (1) Sporadic  (2) Daily | (1) Manual de-identified text (2) Real-time EHR data | (1) Self-taught  (2) Hospital Onboarding | (1) Low  (2) High |
| N25 | (1) DeepSeek  (2) Doubao | (1-2) Personally Accessed | (1) Text polishing & document optimization  (2) Guide search (providing guideline titles for further retrieval), PPT content summarization (course outline generation) | (1-2) Occasional | (1-2) Manual de-identified text | (1-2) Self-taught | (1-2) Low |
| N26 | (1) ChatGPT  (2) iFlytek Spark  (3) DeepSeek | (1-3) Personally Accessed | (1) Text framework organization  (2) Paper structure & formatting  (3) Information retrieval, Medical information reference | (1-3) Occasional | (1-3) Manual de-identified text | (1-3) Self-taught | (1-3) Low |
| N27 | (1) DeepSeek  (2) Doubao  (3) Kimi  (4) Tencent Yuanbao  (5) Risk Prediction System | (1-4) Personally Accessed  (5) Hospital-Integrated | (1) Information integration & synthesis  (2) PPT outline generation  (3) Drug dosage calculation  (4) Cross-disciplinary knowledge retrieval  (5) Auto-calculate venous thrombus embolism risk & prompt prophylaxis | (1-4) Occasional  (5) Daily | (1-4) Manual de-identified text  (5) Real-time EHR data | (1-4) Self-taught  (5) Hospital Onboarding | (1-4) Low  (5) High |
| N28 | (1) DeepSeek  (2) Doubao  (3) Clinical Decision Support System | (1-2) Personally Accessed  (3) Hospital-Integrated | (1) Information integration & synthesis  (2) PPT outline generation, Survey questionnaire framework, Science video learning (AI-assisted production), Daily life predictions/calculations  (3) Medication interaction review | (1-2) Occasional  (3) Daily | (1-2) Manual de-identified text  (3) Real-time medication orders | (1-2) Self-taught  (3) Hospital Onboarding | (1-2) Low  (3) High |
| N29 | (1) DeepSeek  (2) Doubao | (1-2) Personally Accessed | (1) Plan/document drafting  (2) Information retrieval, PPT making, Health education material generation (images, short videos) | (1-2) Occasional | (1-2) Manual de-identified text | (1-2) Self-taught | (1-2) Low |
| N30 | (1) DeepSeek  (2) Doubao | (1-2) Personally Accessed | (1) Drug usage & new technique queries  (2) Knowledge system summarization, Document/PPT improvement suggestions | (1-2) Occasional | (1-2) Manual de-identified text | (1-2) Self-taught | (1-2) Low |
| N31 | (1) DeepSeek  (2) ChatGPT  (3) Doubao | (1-3) Personally Accessed | (1) Text polishing & optimization  (2) Image generation  (3) PPT making, Article framework & idea generation, Chinese-to-English translation | (1-3) Occasional | (1-3) Manual de-identified text | (1-3) Self-taught | (1-3) Low |
| N32 | (1) DeepSeek  (2) Doubao | (1-2) Personally Accessed | (1) Science article writing  (2) Science image generation, Voice/video health education generation | (1-2) Sporadic | (1-2) Manual de-identified text | (1-2) Self-taught | (1-2) Low |
| N33 | (1) Health Management Platform  (2) Intelligent Voice System  (3)Wenxin Yiyan  (4) Doubao | (1-2) Hospital-Integrated  (3-4) Personally Accessed | (1) High-risk population screening & follow-up reminders  (2) notification service  (3) Nursing guideline retrieval  (4) Health education copywriting | (1) Daily  (2) Regular  (3-4) Occasional | (1-2) Real-time EHR data + voice recordings  (3-4) Manual de-identified text | (1-2) Hospital Onboarding  (3-4) Self-taught | (1) High  (2) Moderate  (3-4) Low |
| N34 | (1) Kimi  (2) Risk Prediction System | (1) Personally Accessed  (3) Hospital-Integrated | (1) Clinical knowledge retrieval (unfamiliar conditions, nursing care)  (2) Auto-calculate risk & prompt optimization | (1) Occasional  (2) Daily | (1) Manual de-identified text  (2) Real-time EHR/pre-op data | (1) Self-taught  (2) Hospital Onboarding | (1) Low  (2) High |
