# Supplementary material for: The Double-Edged Sword Effect of AI Application Among Clinical Nurses Based on the Job Demands-Resources Model: Qualitative Study
Source: JMIR Nurs. 2026 Oct 2;9:e100916. doi: 10.2196/100916 (PMC13632912; doi:10.2196/100916)
Supplement: Supplementary file 5 [file nursing-v9-e100916-s005.doc]

### Multimedia Appendix 5. Thematic coding tree based on the JD-R Model

| ****JD-R Dimension**** | ****Theme**** | ****Sub-theme**** | ****Participant quotes**** |
| --- | --- | --- | --- |
| **Job Resources (Gain Path)** | **Workflow Efficiency Leap** | **Documentation Automation** | “Nursing documentation can be generated quite efficiently, which also reduces the time spent on manual handwriting.” (N1)  “When preparing speech drafts, AI can provide some references and help organize an outline more quickly, allowing me to complete the task more effectively.” (N3)  “It helps us nursing staff complete some of the tedious documentation work.” (N5)  “I input the key vital signs, and it expands them into a paragraph that meets documentation standards; this saves me a lot of typing time.” (N7)  “When writing those repetitive documents for medical insurance or quality control... now we have AI voice entry and automatic expansion.” (N7)  “I would say a very plain sentence and then ask it to polish it for me, making it sound more professional or the content more substantial.” (N9)  “For instance, if I want to create an outline myself, it might take a bit longer. But if AI helps me, it only takes one or two minutes, or even just twenty or thirty seconds.” (N10)  “Once, the hospital needed us to give a small lecture. I input the required content into the AI and asked it to create an outline. In less than two minutes, it laid out a perfect framework for the PPT.” (N10)  “I ask it to help me polish a paragraph or modify it to make it more substantial; it makes my written expression much clearer.” (N11)  “When writing summaries—since my writing skills or style aren't that great—I let AI polish them for me.” (N14)  “When writing nursing records... I give the blood test results to the AI to re-edit. It organizes the text and adds the correct units, which is actually very convenient.” (N14)  “I use it to help me polish text or generate some content that isn't strictly professional or rigorous.” (N16)  “I have it generate a first draft for different sections of the content, and then I modify it myself.” (N16)  “It helps with revising papers, writing health education articles, generating PPT outlines, and writing grant proposals.” (N20)  “When writing publicity drafts or creating promotional posters, AI is quite convenient; Doubao is very easy to use.” (N21)  “Our electronic medical record system has introduced AI technology, which can automatically organize and analyze patient records and extract key information, allowing us to understand the patient’s condition more quickly.” (N23)  “After the AI polishes some of the phrasing, I feel there's a significant improvement—it’s like it’s been elevated. The content might be the same, but after it’s processed, the overall feel is completely different.” (N25)  “It helps me create PPTs. Based on the framework I propose, it can quickly integrate resources and provide a foundational structure, making it quite convenient to produce a complete PPT.” (N27)  “My writing ability isn't that strong, especially for documents requiring a specific official format, like government 'red-headed' documents. I look to AI for that, and it provides a systematic plan.” (N29)  “If I have a passage that isn't described very well, it can help me optimize it.” (N31)  “It has integrated AI-assisted entry. During follow-ups for elderly patients with chronic diseases, I speak, and it automatically converts it to text to fill the forms. It can also generate health guidance plans based on the data I input.” (N33)  “Now with AI speech-to-text and automatic form filling... it has liberated us from tedious documentation work.” (N33) |
|  |  | **Task Cycle Reduction** | “It can improve work efficiency, help better regulate nursing workflows, provide standardized operating guidelines, and even offer reminders.” (N1)  “It’s mainly about data organization and summarization because it calculates much faster than we do.” (N1)  “It helps me optimize workflows; for example, it can remind me of medication times or procedures, prioritize tasks, and reduce my overall procedural burden.” (N1)  “It can summarize information from browsers and provide output... allowing you to find answers to questions more efficiently.” (N2)  “It allows me to complete tasks much faster.” (N2)  “It saves me from the step of having to retrieve information myself.” (N3)  “It has saved a lot of energy.” (N3)  “It helps me extract data—specifically, converting my results or R outputs into the data format I want, such as a table or a forest plot.” (N4)  “Without AI, my workload might double. For the methodology section, for example, it takes me a week now; without AI, it might have taken two.” (N4)  “The benefit is that I don’t have to do it myself; it saves me a lot of time and energy.” (N6)  “The AI algorithm can provide early warnings for changing fluids or abnormal flow rates ten minutes in advance based on real-time drip rates and set pharmacokinetic parameters.” (N7)  “I just ask the internal AI, and it lists the mechanism and half-life in seconds. This is a huge help for bedside decision-making.” (N7)  “It also simplifies complex tasks for me.” (N8)  “It saves the time I used to spend searching... by relying on AI for literature retrieval or having it find documents for me.” (N9)  “Using AI to complete tasks within a time limit makes me feel more in control of my workload.” (N9)  “The biggest benefit is that it saves more time.” (N10)  “Creating an outline might take a while on my own, but with AI’s help, it takes only one or two minutes, or even just twenty or thirty seconds.” (N10)  “It is very convenient for querying and integrating information, which has improved my work efficiency.” (N12)  “The biggest benefit is saving time; I feel like I can just use AI to write text that isn't strictly necessary for me to do manually.” (N13)  “It simplifies some operations and doesn't make things more complicated for me.” (N14)  “Searching for resources via AI is indeed much more convenient than doing it ourselves. It saves us a lot of energy in finding resources.” (N15)  “When extracting information from images, it really reduces a lot of workload and makes me feel very relaxed.” (N15)  “It is very convenient, handy, and fast; it can do some work for me so I don't have to go looking for things myself.” (N16)  “Mainly, the preparation time has become shorter than before, which is indeed convenient.” (N18)  “Now that the leadership knows these tools can help, deadlines might be shorter, but my own pressure is also reduced.” (N18)  “Compared to before—like when the head nurse asks for ward rounds or PPT courseware—now it can directly summarize key points for you... it definitely feels more convenient.” (N18)  “Simple, fast, and efficient.” (N19)  “It helps us intelligently assign specific tasks to each person in every group, which reduces time.” (N19)  “It has certainly reduced the time I spend searching for other literature.” (N20)  “The efficiency is very fast, and it’s quite convenient.” (N21)  “It makes the work feel very easy and saves a lot of trouble.” (N21)  “Overall, it saves a lot of time, and it’s very convenient, intelligent, and smart.” (N21)  “It has indeed saved me a lot of time during my work and provided me with much help and convenience.” (N21)  “The biggest benefit is probably finding materials or forming a report more quickly; the efficiency is higher.” (N22)  “The greatest benefit is improved work efficiency... giving us more time to handle other important matters.” (N23)  “The scheduling system arranges shifts reasonably based on our workload and skill levels, allowing us to work in a more planned manner.” (N23)  “The main advantage is that it’s extremely efficient. You just send it your key images, data, and requirements, and it generates the output for you.” (N24)  “It’s much more efficient than searching online yourself and analyzing or comparing things one by one; it’s very fast and convenient.” (N24)  “It has reduced the amount of time I spend.” (N24)  “I feel more efficient and save time... with AI, the time is shortened, efficiency is improved, and the results are quite good.” (N25)  “It saves time—I can only say it saves time. It has really improved my efficiency quite a bit and saved me a lot of time.” (N26)  “If completing a task would normally take half an hour, knowing it only takes 15 minutes with AI gives you a greater sense of control over your time.” (N26)  “I feel it saves time, for example, when making PPTs.” (N28)  “It helped me simplify some things.” (N28)  “In terms of professional knowledge—like writing plans or making schedules—it makes the process faster.” (N29)  “It’s convenient and saves time; it eliminates many unnecessary processes and gives me clear ideas.” (N30)  “It helps me reduce the time spent searching, reading, and summarizing, so I have more time to devote to my actual work.” (N30)  “It’s definitely more convenient than doing it myself.” (N32)  “The biggest benefits are definitely burden reduction and efficiency.” (N33)  “The intelligent voice outbound robot used to notify people about vaccinations or physical exams saves a lot of effort in making phone calls.” (N33)  “It saved me two days of workload.” (N33)  “I can very quickly understand the specific details I want to know, and it's very effortless. If you were to search for it yourself... it would be very time-consuming.” (N34)  “Overall, in terms of duration, it is more concise and allows these tasks to be completed in a short amount of time.” (N34) |
|  |  | **Team Synergy Strengthening** | “During group discussions, we can use AI to organize the questions we need to answer and better establish our future direction.” (N3)  “It allows everyone to have something to contribute during discussions. Everyone can learn a lot through a low-learning-cost path and then discuss it together.” (N4)  “Through AI, we can quickly divide tasks, with each person responsible for a part, while achieving simultaneous information sharing.” (N5)  “When cooperating with the team, it actually accelerates the entire team's progress. If I don't use AI, I feel like I sometimes can’t keep up with the team’s pace at all.” (N6)  “I sent a screenshot of this data to the work group to alert the doctors... It has transformed me from someone who passively executes medical orders into a collaborator who can provide objective data support and proactive warnings.” (N7)  “In teamwork, I am sometimes responsible for part of the information collection, outlining, or PPT creation. I sometimes use AI to search for materials and then share them with other team members who are responsible for different parts.” (N10)  “I sometimes use AI to search for information, and after roughly screening the results, I share them with other team members responsible for other sections.” (N10)  “Using AI combined with my own thinking has improved the sharing and collaboration within the team.” (N13)  “In group learning... we became more cooperative because when discussing a case under tight deadlines, we can use AI to collaborate closely and complete the task within the allotted time.” (N14)  “We use different AIs to generate content, bring it all into a group discussion, and then blend our viewpoints to make the output more complete.” (N15)  “In group learning... we can collaborate closely through AI to complete tasks and course schedules within the required timeframe.” (N16)  “For group cooperation, we can use AI software to help us intelligently assign specific tasks to each person in the group.” (N19)  “When the group needs to complete a task that involves areas no one is familiar with, AI can assist. After the content is co-generated, we learn from it together.” (N19)  “Through the AI system, our nursing team can share patient information in real-time... Every nurse can stay updated on the patient's latest status, avoiding issues with delayed or inaccurate information transfer.” (N23)  “When dividing tasks, AI might be used for personnel allocation or workflow streamlining... If given the chance, I think I would use it because it truly reduces the workload.” (N26)  “Regarding teamwork, I feel it is about cohesion.” (N31)  “Now with the regional medical AI platform, the data I measure for patients in the community can be seen by doctors in large hospitals; the system also automatically captures and pushes discharge summaries from large hospitals to me... Collaboration is much smoother.” (N33) |
|  | **Clinical Cognitive Empowerment** | **Instant Knowledge Replenishment** | “For example, if I want to know what a certain medication is for, I can just seek the information directly; I can use voice input, and it can also give me a voice response.” (N2)  “If I'm not clear about some conceptual information, I can look it up.” (N3)  “I use AI to ask how to perform certain statistics. It’s more than just a search; it actually tells me how the process works.” (N4)  “With AI, you can tell it what you want to do, and it will tell you how to do it.” (N4)  “It possesses a powerful information database. As individuals, there are certainly many aspects we might not think of, but when we utilize it, it effectively supplements our shortcomings.” (N5)  “During operational exams... I can look up specific, detailed steps on it, which helps me make up for my deficiencies.” (N5)  “I use AI to organize and recall anatomical positions and functions; after sorting it out, I have a general idea of the clinical purpose and direction.” (N6)  “Knowledge retrieval for complex vascular surgery medications—vasodilators, anticoagulants, antiplatelets... Now, a quick query to the internal AI provides the mechanism and half-life in seconds.” (N7)  “When I want to search for the effect of something or a drug name, I can find the answer through it just as I would online.” (N8)  “With the help of AI, I can immediately make accurate clinical judgments about what a disease is... it saves me a lot of time in reaching that clinical conclusion.” (N9)  “When I see a drug name, what I use it for most is checking its effects... AI lists everything very clearly, from its mechanism of action and efficacy to its adverse reactions.” (N11)  “It helps me understand some relatively complex theoretical knowledge.” (N12)  “If I don't know the pharmacological action of a drug, I'll ask the AI. It lists the mechanism, efficacy, and adverse reactions very clearly.” (N13)  “It helps me diagnose diseases and write nursing interventions, and I feel that 99% of the information it provides is correct.” (N13)  “Regarding medical knowledge—for instance, asking what happens with a certain disease—I find its answers to be quite accurate.” (N14)  “Through big data retrieval, I can find things that I might not be familiar with.” (N15)  “Through AI analysis, it provides us with many reasons—for example, why a certain clinical manifestation leads to a specific diagnosis. This helps deepen our impression when learning about a disease.” (N15)  “It also supplements my knowledge. Some things from class or textbooks might not be complete, or there might be specific points you care about; you can use it to help refine that relevant knowledge.” (N16)  “The most common use now is equivalent to a Baidu search function. Secondly, when we want to know something more precise, its answers are often more accurate than Baidu's.” (N17)  “I have all sorts of questions. I used to ask Baidu, but now I ask DeepSeek.” (N18)  “Look at my search history—it’s all about various surgeries, different viral infections, and what the symptoms are.” (N18)  “For instance, pharmacological knowledge that I don't quite understand in clinical practice, or the preparation and use of less commonly used chemotherapy drugs.” (N19)  “When preparing chemotherapy ratios, it might involve calculations based on height and weight; it can perform these conversions very conveniently and quickly.” (N19)  “AI is sometimes very detailed when analyzing a patient's lab results or examination reports.” (N21)  “For example, when blood results come out, and I don't understand them, I send them to Doubao and ask it to help me analyze what these results mean.” (N21)  “In clinical practice, if I suddenly want to know a result regarding blood gas analysis, how to use a defibrillator, or the parameter settings for a ventilator... I can understand it quickly.” (N22)  “AI can also provide us with learning resources, such as the latest nursing knowledge and research findings, helping us constantly improve our professional level.” (N23)  “I searched for information once when the head nurse asked about the difference between livor mortis, ecchymosis, and mottling during the morning meeting. It gave a very detailed explanation of each cause and how they form; the explanation was exceptionally thorough.” (N24)  “In terms of understanding pharmacological actions at the level of a general user, I think it performs well.” (N24)  “I'll ask it to see if there are any clinical guidelines. It might list some for me, and based on that, I can then do further searches on websites.” (N25)  “It’s not like a search engine where you only get one aspect of a topic. I feel AI is more comprehensive now; it has an information synthesis function that can give you details from all sides.” (N26)  “It helps me understand professional knowledge from other fields that I’m not very familiar with... especially when you encounter interdisciplinary or combined cases.” (N27)  “I generally use it in clinical practice to check medication instructions or to look up relevant content about new technologies when I encounter them.” (N30)  “It's used for clinical-related drug usage and the application of new clinical techniques.” (N30)  “For example, when I want to acquire certain knowledge, it can provide a very detailed answer.” (N31)  “It has been a huge help in knowledge retrieval... by taking a photo or entering a drug name, it immediately gives me the pharmacological effects and contraindications. It’s like carrying an expert database with me.” (N33)  “When I encounter knowledge I don't understand in the clinic, I end up checking with AI because I find its searches to be more comprehensive and systematic.” (N34)  “If I encounter an unfamiliar term, an unfamiliar disease, or an unfamiliar nursing procedure in clinical practice, I will look it up.” (N34) |
|  |  | **Thinking Logic Scaffold** | “It helps me clarify certain ideas more quickly. For example, when I’m creating mind maps and don’t have a clear direction, it can assist me.” (N2)  “Using the ideas provided by AI, I can analyze this deep learning model step by step—to understand what this convolutional neural network is actually about.” (N2)  “During my studies, it helps me create mind maps and review knowledge.” (N2)  “It can generate a PPT framework based on the content of your documents. If you don't know what the overall structure of a PPT should look like, you can refer to its framework.” (N3)  “With AI, you can tell it what you want to achieve, and it will tell you how to do it.” (N4)  “If I don't know how to start or what method to use for a design, I ask the AI step by step.” (N6)  “I use it to learn and think about how to unfold a project, how to conduct interviews, and how to design my entire workflow.” (N6)  “When I'm stuck on how to approach something, I might need AI to point the way. For example, when writing a report for the first time, I needed AI to give me a logical pivot. It also told me how to structure the logic of the text.” (N8)  “I need AI to give me a logical pivot. It also tells me how to write the logic of a particular passage.” (N9)  “I rely on it to create a detailed mind map—or a table similar to a mind map—so I can better understand the general scope of a knowledge point.” (N9)  “It laid out an excellent framework for a PPT. I just needed to fill in the content I had prepared myself.” (N10)  “Deep thinking. It deeply analyzes the user's question, lists the analytical process in text, and finally forms an answer.” (N11)  “I ask it to point out if there are any errors in my understanding, and then I let it write its own interpretation of the literature. Its depth and breadth of thought are greater than my own.” (N13)  “When making a PPT, I ask it to provide an outline, and then I work based on those main points.” (N14)  “It broadens my horizons. It can suggest angles we might not usually think of; using it to generate an overall overview can expand our perspective.” (N15)  “It can help you organize the connections and context between different pieces of knowledge.” (N16)  “If you give it very accurate instructions, it will show you which directions to take, so you don't have to do too much heavy thinking yourself.” (N18)  “It explores things more deeply. It understands aspects we can't reach, or it shows the multifaceted nature of a topic. Through its analysis, it provides a diversified perspective.” (N19)  “The logic is quite clear.” (N20)  “It helps me improve the logical flow of my articles.” (N20)  “It also tells me which aspects are more appropriate to include when writing a paper, and which angles would make the discussion more effective.” (N22)  “The intelligent diagnostic support system analyzes several possible etiologies based on the patient's symptoms, signs, and test results, and provides corresponding treatment suggestions.” (N23)  “It helps me quickly integrate resources and provides me with a foundational framework.” (N27)  “For survey questionnaires, it provides an overall line of thought for you to follow. It feels like having a clear direction.” (N28)  “I feel that it provides me with a way of thinking.” (N28)  “It can provide inspiration. Sometimes, like when making a PPT and you’re unsure about the layout, it can give you some ideas.” (N29)  “It provides a systematic plan, including organizational deployment, specific implementation stages, and expected results—it can even output all of this in a table.” (N29)  “Bedside blood filtration involves many different techniques, and my knowledge system was quite messy. Every time I encounter something new, I ask AI to summarize and categorize it for me. Now, I clearly understand each technique used in our department.” (N30)  “When writing articles, it provides me with a very clear line of thought.” (N31)  “I might have the seeds of an idea, but don't know how to flesh it out; that’s when I need its help.” (N31)  “When I had to do a popular science promotion and had no ideas, I asked it to help me generate some.” (N32)  “It is simply a tool that provides me with ideas and information.” (N34)  “It influences my initial thoughts; I feel it has a certain guiding quality.” (N34) |
|  |  | **Clinical Safety Safeguarding** | "It can also serve as a risk warning assistant, for example, by indicating the probability of risks such as pressure ulcers, falls, or infections." (N1)  "By utilizing AI to query the original protocols and guidelines, we can potentially avoid these issues." (N5)  "After neurosurgery, what should be paid attention to, and how should care be provided?... When I ask the AI, it tells me the key points to focus on." (N6)  "The VT risk prediction model automatically extracts lab results, surgical records, and bedridden duration from medical records." (N7)  "It’s like a tireless sentinel... It can detect even slight deviations in physiological parameters." (N7)  "It might explain the key points of certain skills. For example, if it’s about giving an injection, it could specify the angle, whether the needle should face upward or downward—those kinds of details." (N16)  "Once, I checked because I thought the doctor’s prescription might have an excessive dosage for a child, so I searched online briefly." (N18)  "For things like chemotherapy drug preparation, it’s quite reliable—it usually pulls the latest literature... or if you look up wound care or international guidelines, it should provide the most up-to-date information." (N19)  "Preparation methods, how long the drug should be used, whether additional monitoring is needed—things like that." (N19)  "In the gastroenterology department, they have a nutrition screening machine that automatically scans body composition measurements, and we directly adopt its results." (N21)  "The intelligent early warning system mainly monitors patients’ vital signs... If any data is abnormal, it immediately alerts us so we can respond quickly." (N23)  "The risk prediction model can forecast the likelihood of certain complications based on patients’ indicators, allowing us to take preventive measures in advance." (N23)  "For clinical tasks, yes—for example, verifying medication dosages... Yes (the results are accurate and timely)." (N27)  "For tasks like documentation or reviewing orders, it can help us identify and filter out errors." (N30)  "The system suddenly popped up a red alert, indicating extreme blood pressure variability and a high short-term risk of stroke." (N33) |
|  | **Professional Capital Appreciation** | **Emotional Energy Replenishment** | "Even though it’s complicated, we still have AI, right?... It boosts my confidence in my work. With that confidence, I’m less likely to be bothered by difficulties." (N4)  "Using it improves my work efficiency. In that sense, it motivates me and keeps me from feeling burned out." (N6)  "Overall, it’s more positive." (N8)  "AI helps me complete tasks within the allotted time, so I feel like I’m saving energy in that area. Then I can channel my energy into other things, making me feel more energetic overall." (N9)  "I think it provides positive feedback." (N14)  "I’d say the former (referring to feeling more energetic and focused)." (N18)  "A good companion... it offers me a lot of help." (N18)  "More energetic." (N21)  "Because this AI tool exists, my resistance to complex data analysis decreases." (N22)  "Chatting with it helps relieve boredom... After venting, it offers rational and even humanistic perspectives—I think its analysis is quite good." (N22)  "Overall, it’s positive." (N26)  "When I needed it, it provided me with meaningful support." (N27)  "(Using AI makes me feel) more focused and engaged at work." (N30) |
|  |  | **Cross-boundary Skill Expansion** | "It can help me learn certain skills." (N2)  "This belongs to the field of computer science, which might be particularly challenging for us because it involves many complex algorithms. So, we rely on AI to assist us." (N2)  "Learning software, data analysis, or Python—if we have AI, it can genuinely help enhance these abilities." (N4)  "It actually expands my knowledge. It doesn’t just answer my immediate question; it extends my inquiry into broader aspects." (N6)  "I can revisit and reinforce my understanding of the knowledge I need to master." (N6)  "The ability to construct precise prompts has become a new implicit skill in nursing work." (N7)  "To prove it wrong, I had to master the diagnostic criteria more thoroughly." (N7)  "Through AI-assisted training, I achieved my goal." (N9)  "It deepened my understanding of clinical medication." (N11)  "For example, AI can now edit photos. If some images are difficult to retouch manually, AI can enhance them." (N12)  "It assists in analyzing imaging data or wound assessments. It provides insights to help resolve current dilemmas." (N19)  "In research, it might involve methodological tools, such as SPSS operations or selecting appropriate statistical methods." (N22)  "It improved my clinical knowledge and techniques (e.g., blood gas analysis, defibrillator use)." (N22)  "I’ve had to learn a lot about statistics and data analysis, which broadened my expertise." (N23)  "Even while using AI, I’m simultaneously learning from its outputs—analyzing and adopting new techniques." (N25)  "I’m currently learning to use AI to create short educational videos, though I haven’t fully figured it out yet." (N28)  "My writing skills aren’t that strong... It generates a systematic plan... tailored to my institution’s actual conditions, incorporating relevant elements." (N29)  "In dermatology and cosmetology, they use AI to create a virtual nurse avatar for patient education. I think this is a great idea." (N29)  "This is an added benefit—it undoubtedly equips me with an extra skill." (N32)  "It forced me to learn data analysis. Before, I just followed orders; now, I interpret AI-generated reports and analyze chronic disease trends in my district... It transformed me from an executor into a manager." (N33) |
|  |  | **Core Value Regression** | "It can take over more mechanical tasks, such as documentation or monitoring, allowing nurses to focus more on patient psychological care and complex clinical management." (N1)  "It frees our attention from continuous, passive scanning, redirecting it to more valuable patient assessments and communication." (N7)  "I hope AI can truly automate tedious tasks... If achieved, nurses could return to patients’ bedsides to do what only humans can." (N7)  "I expect it to reduce paperwork for on-duty nurses... minimizing repetitive tasks." (N9)  "If AI could compile required data or daily rounds records... and input them into the system, wouldn’t that reduce nurses’ workload and make their jobs easier?" (N10)  "I hope it can standardize procedural aspects of our work... But compared to humans, AI still lacks humanistic care." (N12)  "It could reduce repetitive physical labor—for instance, making beds for us." (N13)  "Tasks like double-checking medical orders could actually be resolved by AI." (N14)  "For documentation or equipment-related tasks, AI’s assistance is significant." (N15)  "I hope... it decreases nurses’ clinical workload while improving patient care quality and safety." (N20)  "I hope simple tasks like discharge education or admission guidance could be handled by AI." (N21)  "I want AI to lighten our workload so we have more time to care for patients." (N23)  "I hope... AI can play a role for nurses, providing patients with health education and some popular science information." (N26)  "It saves documentation time... giving us more opportunities for direct patient interaction." (N33)  "Using AI to process data, then spending the saved time holding an elderly patient’s hand or chatting." (N33)  "I want it to generate personalized nursing plans for patients... offering nursing insights, diagnoses, and interventions." (N34) |
| **Job Demands (Drain Path)** | **Cognitive Impediment** | **Verification Overload** | "In individual diagnosis and treatment-related aspects, the results can vary significantly... We still need to cross-check with clinical experience or other case studies." (N1)  "It generates fake references—either attributing them to non-existent authors or inventing studies... forcing me to search myself again." (N2)  "While it helps structure ideas, it fabricates references." (N2)  "For greater accuracy, you must verify its outputs a second time." (N3)  "You need to double-check the information it generates." (N3)  "It compiles tables you can copy-paste directly, but they still require verification—though it’s faster than manual entry." (N4)  "If you ask AI to generate references, they’re fake... You must read them yourself to assess relevance. This adds unnecessary work." (N4)  "Data may be outdated due to delayed updates, reducing accuracy and requiring extra scrutiny." (N5)  "I often have to re-search information, review key points, and revise AI-generated results." (N5)  "Sometimes the information is overly repetitive. While it saves time, filtering duplicates affects efficiency." (N6)  "But ultimately, its correctness must be verified by us." (N6)  "Getting results from AI is easy—trusting them isn’t. This forces nurses to add a manual correction step." (N7)  "Time spent auditing, editing, and validating sometimes exceeds writing from scratch." (N7)  "If colleagues submit AI-generated work without review... you must sift through, modify, and supplement it." (N8)  "It makes errors in simple calculations... The output feels artificial, so manual validation is inevitable." (N9)  "Fabricated content forces me to review everything, draining time and energy." (N9)  "Over half the references it provides are fabricated—I have to re-find them myself." (N10)  "I must screen the data again to isolate usable elements... Otherwise, errors may propagate." (N10)  "Interventions might alter patient records or meeting notes... Nurses then bear the burden of correcting misinformation." (N10)  "I find it unreliable. It cites outdated materials—like 2010s data in 2025." (N11)  "Accuracy gaps require cross-referencing literature, paradoxically increasing workload." (N12)  "Information overload necessitates filtering irrelevant content... followed by rechecks." (N12)  "Spotting one error means re-validating the entire output." (N13)  "Secondary verification is exhausting." (N13)  "Some outputs clearly deviate from source materials." (N13)  "Tools like DeepSeek or Wenxin generate non-existent references." (N14)  "You can only borrow its logic—many details are flawed." (N14)  "If the output seems implausible, I recalculate everything manually." (N14)  "Most AI-generated references are fake... I re-search each title, adding hidden labor." (N15)  "I replace content by consulting actual literature and resources." (N15)  "The downside? Questionable accuracy... requiring independent checks." (N16)  "Documents demand rigor—I verify every AI claim individually." (N16)  "You must still judge accuracy yourself." (N17)  "For academic precision, I always conduct my own searches." (N17)  "Outputs vary across tools... I re-research to confirm correctness." (N18)  "Language remains stiff, requiring rewrites." (N20)  "I ignore its data/references—only its logic is useful." (N20)  "Manual edits are needed to fix inappropriate terms." (N21)  "Medical illustrations it generates often lack precision, necessitating replacements." (N21)  "Errors are perceptible—I demand rechecks when it delivers false information." (N22)  "Three out of five references are fabricated, forcing supplemental searches." (N22)  "Discrepancies between AI and clinical judgment waste time troubleshooting." (N23)  "Specialized topics require literature reviews—their answers are incomplete." (N24)  "I cross-check since it’s not consistently accurate." (N24)  "Fabrications are rampant... It invents plausible author/journal details." (N26)  "Blind trust is impossible—outputs require active vetting." (N26)  "Post-search, I find no trace of its cited 'sources'... It’s demoralizing." (N26)  "Separating fiction from facts relies on personal expertise." (N27)  "Data may be crowdsourced or manipulated—discernment is essential." (N27)  "Content requires tweaks as details often miss the mark." (N28)  "References may be inaccurate or entirely fabricated." (N29)  "The burden lies in judging correctness... prompting follow-up searches." (N29)  "In 15 references, 5-8 might be fake." (N29)  "I verify reference existence and content." (N30)  "Information vetting is a tedious multi-step process." (N30)  "Some outputs are incomplete or inaccurate, demanding re-research." (N31)  "Using its results still requires verification—it’s not truly effortless." (N31)  "I never use its references without revalidation." (N31)  "The biggest flaw? Mandatory rechecks to prevent errors." (N32)  "Avoiding duplicate work means adding verification steps." (N32)  "Data cleaning is the main pressure point... It merges records of same-name patients or mislabels deceased ones as 'needing follow-up,' requiring hours of corrections." (N33)  "Personalized inaccuracies occur... AI can’t fully grasp individual contexts, leading to deviations." (N34)  "Confirming reference validity consumes energy." (N34) |
|  |  | **Interaction Fatigue** | "Initially, when giving instructions to AI, it often didn’t understand my intent, requiring constant refinement." (N5)  "Ambiguities inevitably arise, necessitating repeated questioning—this creates early-stage burdens." (N5)  "Sometimes, even after rephrasing multiple times, I still can’t get the desired output." (N6)  "Learning new tools always involves hurdles... There’s inevitably some initial friction." (N6)  "I waste three minutes arguing with it to prove my clinical judgment is superior." (N7)  "Once, the system alerted 'Bed 3 emergency'—I rushed over only to hear 'False alarm, AI error.' My temper flared instantly." (N7)  "Inputting prompts, waiting for responses... The process feels draining when I’m deeply invested." (N13)  "It rarely aligns with my vision—I exhaust myself explaining how to adjust the output." (N16)  "The initial workflow isn’t smooth. You must learn its quirks—which terms trigger better responses." (N17)  "Results hinge on phrasing, demanding iterative tweaks." (N20)  "Submitting error reports, waiting for AI adjustments—this cycle is tedious." (N22)  "What I assumed would take 5-10 minutes sometimes stretches to 2-3 hours for high-precision images... Debugging feels absurd—like it doesn’t comprehend human language." (N22)  "Frequent system glitches or false alarms spike frustration and mental strain." (N23)  "It disrupts workflows, forcing us to drop tasks for unnecessary checks." (N23)  "We feel puppeteered by its incessant alerts." (N23)  "When outputs miss the mark, I rephrase prompts or feed it more context—a tiring loop." (N26)  "Initial questions lack precision, requiring AI-guided refinements." (N27)  "It often misses the crux of queries, responding too generically." (N28)  "You invest extra time vetting its correctness—another hidden cost." (N29)  "Secondary verification is exhausting... The filtering process is overly cumbersome." (N30)  "Getting irrelevant results is infuriating." (N31)  "Does re-searching demand energy? Absolutely." (N31)  "Some generated images require multiple attempts to match expectations." (N32)  "If outputs are wrong, rework time balloons." (N32)  "The system’s rigid workflow forces compliance—I’ve become an algorithm’s puppet." (N33)  "The learning curve is steep... Even creating PPTs through AI feels like a negotiation." (N34) |
|  |  | **Mental Dependency** | "Long-term reliance on AI for monitoring or documentation may erode our proficiency in core tasks." (N1)  "Overdependence on AI conclusions could weaken nurses’ clinical judgment, leading to misdiagnoses of unique patient conditions." (N1)  "The downside is it disrupts one’s thought process... Excessive AI use breeds dependency, overshadowing my original reasoning." (N2)  "It also makes me personally lazier." (N2)  "I unconsciously default to AI for task completion, bypassing independent thinking—this gradual reliance dulls cognitive engagement." (N3)  "I feel like I’m relinquishing critical thought—a net loss for human capability." (N3)  "Admittedly, excessive reliance develops. For instance, I now struggle to work without internet access." (N4)  "Without AI, tasks become arduous... The dependency is too entrenched." (N4)  "Dependency is a real risk. Prolonged use of convenient tools fosters mental laziness." (N5)  "Initially, I took search results at face value without scrutiny... AI dominated my workflow." (N6)  "I depend on its alerts yet distrust its fabrications—this tension is more draining than pure fatigue." (N7)  "Junior nurses blindly trust system outputs, corroding their clinical reasoning over time." (N7)  "The most obvious issue is dependency. People stop exerting effort independently." (N8)  "Extended use leads to rigid thinking... Self-completion would bring a greater sense of achievement and growth." (N8)  "AI risks making people lazy." (N10)  "Its convenience discourages autonomous task completion—we default to AI solutions." (N10)  "The drawback is how easily modern humans over-rely on it." (N11)  "I worry about becoming overly dependent myself." (N11)  "My greatest concern is societal overreliance—that’s the real danger." (N11)  "Overdependence dulls my initiative, stifling creativity and original thought." (N12)  "The worst part? Mental atrophy—prolonged AI use makes my brain feel sluggish." (N13)  "Without AI proofreading, I’d second-guess my spelling... Now I don’t even check." (N13)  "Sometimes I’ve surrendered critical thinking—it’s habitual now." (N14)  "It confines thought within narrow parameters, eroding our ability to broaden perspectives." (N15)  "Previously, I’d design workflows independently... Now my first instinct is to ask AI—dependency deepens." (N15)  "It breeds burnout. Reliance suppresses problem-solving motivation." (N15)  "After using it, I realize I’ve gained no real skills." (N16)  "Divergent thinking diminishes... I feel it happening." (N16)  "The negative impact outweighs benefits—it fuels my lethargy." (N16)  "Dependency grows—I’ve stopped consulting literature... just gotten lazier." (N17)  "The biggest downside is inertia. Tasks that once demanded thought now require minimal effort." (N18)  "Opportunities for active reasoning shrink. AI handles everything—independent thinking atrophies." (N19)  "My fear? Widespread AI adoption will degrade our cognitive capacity, making us complacent." (N19)  "Its frameworks diverge from mine... Yes, they constrain creative ideation." (N20)  "While helpful, its suggestions can override my original conceptualization." (N20)  "It enables laziness—why brainstorm when AI generates ready-made outputs?" (N21)  "Personally, I don’t feel constrained—but I’ll admit I enjoy cutting corners." (N21)  "I’ve developed passive thinking. My first reflex is to consult DeepSeek or GPT... Innovation suffers." (N22)  "Overreliance degrades our core skills and judgment—that’s the real cost." (N23)  "You just stop engaging your brain." (N24)  "I’ll lazily grab a premade summary from AI... but it doesn’t fundamentally impact me." (N24)  "Prepackaged solutions breed dependency... Mental muscles weaken without exercise." (N25)  "Prolonged use creates reliance—certain abilities deteriorate from disuse." (N26)  "It shortcuts my thought process, stripping fulfillment from results." (N30)  "My unexercised skills have atrophied." (N31)  "Thought becomes boxed within preset frameworks... absolutely." (N31)  "The downside? Its overly specific outputs limit my broader understanding." (N34)  "I fear growing so adept with AI that my own faculties decay." (N34) |
|  | **Relational Attrition** | **Interpretive Labor** | "Now, many patients come to consultations armed with AI-generated information to question doctors... When AI outputs conflict with medical explanations, doctors spend extra time clarifying. This undoubtedly increases their workload." (N3)  "When patients notice discrepancies between AI content and doctors’ advice, they grow anxious. They persistently seek reassurance, forcing doctors to invest significant time explaining the rationale." (N3)  "Families often confront me with smartphone screenshots... These situations demand exhaustive explanations, compounded by their AI-fueled fears." (N7)  "Patients self-diagnose via AI searches... As nurses, we must look beyond surface-level queries and redirect them to evidence-based textbook knowledge." (N11)  "Sometimes, when families ask questions I can’t immediately answer, I discreetly research first, verify with senior staff, then re-engage—a time-intensive process." (N13)  "Patients and families lack AI literacy... We expend effort demystifying its mechanisms to alleviate their concerns." (N23)  "Families obsess over infusion pump accuracy, fearing malfunctions—we patiently walk them through the safeguards." (N23)  "Colleagues sometimes debate AI-derived treatment plans, requiring additional coordination to reach consensus." (N23)  "During outreach clinics, patients cross-reference AI findings with us—it’s becoming routine." (N25)  "Clarifications are non-negotiable labor—no one pretends this burden doesn’t exist." (N25)  "Patients and families create challenges by consulting unreliable online sources... This undermines our health education efforts." (N30)  "We must vet information; they lack that skill. So we reiterate explanations endlessly." (N30)  "AI offers generic advice, while we provide personalized care plans—this mismatch necessitates constant re-teaching." (N30)  "Elderly patients frequently ask: ‘Nurse, why does the robot caller sound strange?’ or ‘The system says I’m diabetic, but I feel fine!’ Calming such AI-related anxieties consumes hours." (N33)  "What should be simple nurse-patient dialogues now require machine literacy lessons—communication overhead balloons." (N33)  "Beyond standard medical explanations, we must dissect why AI’s outputs may not apply to their specific case—a dual-layered time drain." (N34) |
|  |  | **Care Dehumanization** | "It tends to overlook patient individuality—special physiological signs or psychological states, anything nuanced." (N1)  "Lacks the depth of clinical experience and humanistic care skills." (N1)  "Nursing is fundamentally human-to-human interaction, not machine-to-human transactions—this risks stripping away warmth and empathy." (N5)  "I have to stare at the PDA screen. Patients think I’m just playing with my phone. These interruptions erode trust in nurse-patient relationships." (N7)  "When the PDA incessantly buzzes, demanding I change an IV bag next bed, it reduces me from a compassionate caregiver to a task-executing robot." (N7)  "Compared to humans, it’s deficient in humanistic care—a cornerstone of our profession." (N12)  "Could AI compromise privacy—ours or patients’? That’s a critical concern." (N15)  "My greatest fear? The inability to empathize... AI is fundamentally different from humans." (N19)  "Patient acceptance matters. Some distrust or fear AI due to limited understanding." (N23)  "It may compromise humanistic care or data accuracy, endangering patient safety." (N24)  "Medicine deals with lives—its rigor is non-negotiable. AI’s reliability remains debatable." (N26)  "Diagnosing via AI risks overstatement... Like when patients self-search symptoms and get misled into believing they have cancer." (N28)  "Nursing isn’t just knowledge-based... AI can’t physically assess a patient—take pulses, observe fever patterns." (N30)  "The downside? It’s impersonal and rigid... Over-reliance turns nursing into a soulless checklist." (N33)  "If patients input subjective data, AI may miss contextual clues... overlooking alternative diagnoses." (N34) |
|  |  | **Professional Alienation** | "My concern is whether AI might replace this profession entirely." (N2)  "If a patient has muscle issues and you immediately pull out your phone to search, it makes you appear highly unprofessional." (N3)  "AI’s overdeveloped capabilities lead to excessive reliance among clinical nurses, detaching them from practical care realities." (N3)  "Junior nurses, mechanically following AI directives, lose independent observation and reasoning skills—reduced to mere task-runners, AI’s hands and feet." (N7)  "It inevitably breeds patient distrust, and some senior nurses struggle to adapt." (N8)  "Since AI mixes in false information, it could distort medical judgments or patients’ self-assessments, exacerbating doctor-patient conflicts." (N10)  "If it outperforms humans, could nurses become obsolete?" (N13)  "Clinical settings are unpredictable... Some scenarios demand immediate experienced judgment—human presence remains irreplaceable." (N14)  "My greatest fear is AI wholly supplanting nursing roles, eliminating job opportunities." (N23)  "If a patient deteriorates, doctors must still assess the situation first... AI’s authority feels inadequate." (N28)  "The core concern is its lack of authoritativeness." (N28)  "Total AI dependence fractures teamwork... that’s the perception." (N31)  "I dread nursing becoming purely screen-bound, stripping away our essence—human compassion and warmth." (N33) |
|  | **Digital Involution** | **Performance Inflation** | "Stop tying AI alert response rates to performance metrics! Once KPIs are linked to AI alerts, nurses will dismiss warnings just to meet targets." (N7)  "AI was supposed to reduce our workload, but it’s become a bottomless pit. For instance, the system auto-recommends care pathways—but if you accept them, it demands meticulous documentation at every step. Miss one item, and your quality score drops. What used to take 5 minutes for a nursing note now requires at least 15 minutes to fulfill all system requirements. Performance metrics appear more precise, but our time is silently consumed." (N7)  "Early on, some colleagues adopted AI while I avoided it. Back then, I noticed my manually written health education materials couldn’t match AI-generated quality." (N15)  "Indeed, at the very beginning. (When there is a task to complete, and you could have chosen not to use AI, but seeing your peers all using AI creates a sense of competition, which increases pressure)." (N16)  "Before, our health education drafts were ‘good enough.’ Now, with AI, expectations keep rising—output must be flawless, especially for WeChat publications. The competition is exhausting." (N23)  "Management increasingly judges quality by numbers rather than substance. Quantity gradually overshadows quality focus, and performance benchmarks keep escalating." (N33) |
|  |  | **Competitive Perfectionism** | "Everyone basically uses AI, and everyone is striving for a state of perfect completion. If I don't use AI, I probably won't achieve good results." (N5)  "Indeed, at the very beginning. (When there is a task to complete, and you could have chosen not to use AI, but seeing your peers all using AI creates a sense of competition, which increases pressure)." (N16)  "For example, when I make a video, the requirements might be higher, and I would want it to look more refined." (N17)  "That is, while it provides convenience, because it exists, your standards become higher." (N17)  "It's quite competitive. Anyway, after AI appeared, everyone became more competitive." (N17)  "I have to spend time after work every day learning related knowledge, and I feel a lot of pressure." (N23)  "Before, when we wrote health education materials, 'good enough' was fine. But now with AI, the requirements keep getting higher, the quality keeps improving — it's very competitive. It's even harder if we want to publish on WeChat public accounts." (N23)  "There might be a little pressure... Because whenever I create something, I want to use AI to check it, and then I make the final decision on whether to use it." (N31)  "It kind of gives me a bit of a perfectionist tendency." (N32) |
|  |  | **Workload Expansion** | "The system pushes real-time patient data—whether I’m eating or in the restroom, my phone buzzes, and I have to check." (N7)  "Nurses haven’t had responsibilities reduced by AI; instead, we’ve gained the added duty of monitoring AI for malfunctions." (N7)  "To feed AI prediction models, we need hyper-precise input data... I must log pain locations, intensity, and characteristics on the PDA." (N7)  "What used to be a three-step process now becomes five steps when AI ‘assists’ with decision-making." (N16)  "It creates invisible pressure—tasks I could’ve skipped now feel mandatory." (N17)  "I feel it’s added unnecessary burdens." (N17)  "While AI boosts efficiency, it demands time to learn, master, and constantly monitor—all to ensure data accuracy." (N23)  "The interface is overly complex, requiring excessive data entry. The multi-step process eats into our time." (N23)  "Being a new technology, there’s inevitably a steep learning curve." (N27)  "Learning it did bring some initial stress." (N27)  "It introduces superfluous elements that I then have to manually prune." (N31)  "The double-entry problem... We’re required to maintain paper records or re-enter data in legacy systems. This means juggling patient interactions with endless screen-tapping—it disrupts care rhythms." (N33)  "Software updates bombard us monthly... Forced to relearn interfaces mid-shift—it’s mentally draining." (N33)  "It inflates my workload. Hospitals keep discovering new AI applications, demanding ‘precision refinements’ that pile on tasks—that’s my concern." (N34) |
| **Moderating Mechanisms (Buffering Factors)** | **Proactive Coping Strategies** | **Critical Verification Execution** | "Ultimately, patient care is delivered based on actual clinical experience." (N1)  "All AI-generated outputs—care plans, data results—require secondary validation against patient conditions and clinical expertise." (N1)  "I cross-reference specialized terminology using academic literature or search engines to ensure accurate understanding." (N2)  "I avoid AI-generated references and conduct independent searches." (N2)  "I delve deeper, sourcing detailed materials to verify the authenticity of AI-produced content." (N3)  "Vigilant discernment is non-negotiable." (N3)  "Research design and coding remain my domain—AI merely assists." (N4)  "Full reliance on AI for coding introduces undetected flaws. Manual review is essential." (N4)  "I never parrot AI outputs verbatim. They serve as references, but clinical mentors are my primary consult." (N6)  "If findings contradict my foundational knowledge, I reevaluate and reassess AI’s conclusions." (N6)  "My protocol: AI alert → sensory verification → interference filtering → final judgment." (N7)  "AI’s propensity for fabricated data demands rigorous screening." (N10)  "AI lacks the rigor for this issue—I default to textbooks." (N11)  "I first develop independent frameworks, then use AI to supplement—not supplant—my reasoning." (N12)  "When patients question AI outputs, I lead with established theory, then corroborate with evidence." (N12)  "After consulting senior nurses for verification, I re-engage families." (N13)  "Triangulating outputs across multiple AI platforms." (N13)  "I recalculate everything manually." (N14)  "Accuracy emerges only when you layer AI outputs with independent analysis." (N14)  "I distrust its data—it’s relegated to drafting outlines; I substantiate content and verify." (N15)  "Cross-comparisons via disparate AI tools." (N15)  "Every claim requires individual vetting." (N16)  "Growth stems from self-reliance—AI is just a support tool." (N16)  "Serious scholarship demands personal literature reviews—AI alone yields fragmented perspectives." (N17)  "Suspecting inaccuracies, I investigate discrepancies exhaustively." (N18)  "Deploy tools judiciously—only after exhausting independent problem-solving." (N19)  "AI’s immaturity warrants skepticism—I never fully trust its data." (N20)  "For high-stakes decisions, academic sources trump AI every time." (N21)  "AI drafts undergo literature validation for reliability." (N21)  "Results always require manual auditing—blind trust is reckless." (N22)  "I refine, modify, and adjust—never deploy AI outputs raw." (N22)  "AI conclusions are filtered through professional judgment—never adopted uncritically." (N23)  "Specialized domains demand my own eyes—AI doesn’t earn that trust." (N24)  "Current stance: multi-source verification precedes acceptance." (N26)  "Reality-checking against known knowledge." (N27)  "Evidence-based work excludes AI—it lacks the rigor." (N28)  "I interrogate references for existence before adoption." (N30)  "AI’s role? A thinking aid—used with extreme caution." (N30)  "Trust is partial—50% reference, 50% verification." (N33)  "Knowing AI’s limits prevents blind allegiance." (N33) |
|  |  | **Strategic Interaction Mastery** | "Ensure the accuracy of uploaded or processed data—output quality hinges on input precision." (N1)  "I explicitly instruct it not to fabricate." (N2)  "Identify AI’s strengths and deploy it selectively." (N3)  "Learn to choose the AI best suited for your specific task." (N3)  "You must train it step-by-step... Feed it a model document, and it’ll generate stylistically consistent forest plots." (N4)  "Cost-effective approach: Provide a reference document and command, ‘Emulate this for my version.’" (N4)  "Prompt: ‘For vascular surgery patients post-lower-limb stenting, draft clopidogrel administration guidelines’—it delivers near-ready copy." (N7)  "Local deployment allows custom data training to enhance accuracy." (N10)  "Exercise restraint—avoid overuse. Don’t consult it for everything." (N11)  "Develop independent frameworks first, then use AI for refinement—not wholesale completion." (N12)  "Precise prompts yield targeted results. Specify formats upfront." (N13)  "If the prompt lacks depth, I augment it with critical details." (N14)  "Adapt the framework—supplement gaps yourself." (N14)  "Command refinement is key—iterative tweaks align outputs with needs." (N15)  "Amplify essentials; prune redundancies." (N16)  "I’ll handle the edits myself." (N16)  "Targeted usage matters—know which tool excels at what." (N17)  "Maximize utility by defining search parameters crisply—clarity begets precision." (N18)  "Pre-research topics via literature, then task AI with synthesis." (N19)  "Demand granular specificity in instructions." (N20)  "Hybrid AI deployment—blending multiple tools—optimizes outcomes." (N22)  "Train it by feeding relevant literature... Guide it stepwise through iterative feedback." (N22)  "Parameter tuning requires continuous experimentation and reflection." (N23)  "Articulate the desired output’s blueprint—input core concepts verbatim." (N25)  "Precision in questioning minimizes ambiguity and errors." (N26)  "Sharpen your query’s parameters—the tighter the constraints, the better the analysis." (N28)  "Master command engineering—detailed prompts with exemplars (when uploadable) anchor expectations." (N29)  "Stress-test outputs across multiple AI platforms." (N31) |
|  | **Professional Boundary Demarcation** | **Role Positioning Clarity** | "First, clarify AI’s role—it’s a support tool, not a replacement for human judgment." (N1)  "It’s a high-efficiency aide, not a substitute." (N1)  "Treat it as a reference tool—never offload entire tasks to AI." (N3)  "Like a magnifying glass: it enhances details, but you can still see without it." (N3)  "Primarily, it’s just a tool." (N6)  "An encyclopedic yet non-prescriptive resource—like senior peers who won’t spoon-feed answers." (N6)  "Don’t treat it as authority. See it as a hyper-memorizing but emotionally inept intern—requiring constant feedback and training." (N7)  "An occasionally disruptive mechanical colleague." (N10)  "My little daily helper." (N11)  "I view it more as a tireless apprentice." (N12)  "Just a supportive instrument to refine my work." (N12)  "A beast of burden—diligent but uncomplaining." (N13)  "An assistant, plain and simple." (N14)  "Closer to a supercomputer—ultimately, still just a tool." (N15)  "A decent mentor." (N16)  "A good partner." (N18)  "A collaborative coworker." (N19)  "An auxiliary work tool." (N20)  "More like a partner—one who assists but remains under my direction." (N21)  "A highly efficient, highly specialized secretary or partner." (N22)  "AI resembles a bright but unreliable aide." (N23)  "An unseasoned teacher—it offers insights, but they’re not always correct." (N24)  "A helpful aide, but only a supportive one... It can’t dominate decision-making." (N25)  "A textual assistant." (N26)  "For reference and support—nothing more." (N26)  "AI plays a supporting role—final authority always rests with humans." (N27)  "Secretarial or assistant-tier." (N28)  "A well-meaning but sometimes troublesome confidant." (N29)  "An assistant—though not yet fully integrated into workflows." (N30)  "A collaborative partner." (N30)  "A tool to polish my work." (N31)  "An aide." (N31)  "That tireless apprentice again." (N32)  "It’s like a freshly graduated intern—theoretically strong but socially clueless... So I, as the mentor, must constantly supervise." (N33)  "Just a minor helper—neither teacher nor oracle. The buck stops with me." (N34) |
|  |  | **Clinical Agency Retention** | "Selectively deploy it—prioritize standardized tasks while avoiding individualized diagnostic decisions." (N1)  "You must maintain personal oversight." (N3)  "I preserve autonomous judgment... Ultimately, I control my workflow without feeling overpowered." (N4)  "Nursing expertise remains paramount... For clinical realities, we must strengthen core competencies." (N5)  "I adhere to my own logic—AI won’t override my reasoning." (N6)  "Critical thinking is non-negotiable. With independent thought, AI becomes a tool, not a puppeteer." (N6)  "Master the disease better than AI does—only then can you command it, not vice versa." (N7)  "Dismissing AI alerts sometimes reflects advanced clinical judgment, not negligence." (N7)  "I retain control—dependency hasn’t taken root." (N10)  "Some things demand independent analysis." (N11)  "Clinical decisions require utmost rigor... AI’s reliability is comparatively weaker—I’d never rely on it for judgments." (N12)  "Core work requires self-direction and verified sources—never AI alone." (N12)  "I’d never use this (AI) for clinical judgments." (N17)  "While AI displaces certain tasks, nursing’s essence remains irreplaceable—though some aspects are vulnerable." (N18)  "But these can’t truly substitute human work." (N18)  "Competence stems from personal cultivation—extensive reading, literature synthesis, and research agility matter most." (N20)  "I’d never risk clinical use—only personal queries... But never with patients." (N21)  "Nurses’ professional knowledge and skills take precedence... Clinical realities demand heightened capabilities." (N23)  "For teaching materials, I always create independently." (N24)  "Final authority stays human... Otherwise, agency erodes." (N27)  "Never outsource decisions to it." (N28)  "At best, it aids judgment—but nurses and doctors must assess based on actual patient contexts." (N30)  "I draft frameworks first, then task AI with refinement—it never dictates content." (N31)  "My rule: humanity over machinery. If AI disrupts patient connections, I sideline it." (N33)  "Just a minor assistant—neither teacher nor oracle. The buck stops with me." (N34) |
|  | **Organizational Support Architecture** | **Systemic Resource Provision** | "Hospitals could consolidate databases... Institution-led AI platforms would streamline retrieval." (N3)  "Could we establish clinical informatics nurse roles dedicated to supporting frontline staff?" (N7)  "I’d welcome a hospital-tailored AI system." (N8)  "Medical AI tools should be hospital-subsidized or collectively purchased for free staff access." (N13)  "Free vs. paid versions differ significantly in utility... Hospitals should cover premium costs." (N15)  "Enable access to advanced AI tool accounts." (N20)  "Invest in high-tier AI software for staff-wide distribution." (N21)  "Hope departments could procure premium platforms... and train specialty-specific AI models." (N22)  "Streamline AI-related workflows by eliminating redundant steps." (N23)  "Funding for institutional memberships, that sort of thing." (N24)  "For instance, hospitals could license versions for our use." (N29)  "Paid institutional access would be ideal—enhancing both work and learning." (N30)  "Seek departmental or administrative funding for AI memberships." (N31)  "Essential—no out-of-pocket expenses for staff." (N32)  "Error tolerance mechanisms: clear liability waivers for AI misjudgments, not default nurse blame." (N33) |
|  |  | **Capacity Building Support** | "Organize training sessions on how to accurately and correctly utilize AI." (N1)  "If hospitals adopt AI, they should develop structured teaching programs." (N3)  "If training is mandatory, it should focus on how to properly 'feed' data into AI systems." (N4)  "Participation in formal training is essential—understanding AI’s underlying mechanisms and logic is critical." (N5)  "Even informal discussions about AI usage would be beneficial." (N6)  "Training shouldn’t just cover button-clicking! Teach the logic behind AI and its common failure points." (N7)  "Training should address how to refine AI queries for more accurate, comprehensive outputs and minimize error rates." (N10)  "Guide me on integrating AI without disrupting clinical workflows—not just for medications, but also surgical processes." (N11)  "Train us on AI interaction—how to phrase questions and verify results." (N13)  "Advanced AI applications require dedicated learning." (N14)  "Training should include how to query AI and validate its responses." (N16)  "We previously attended an informatics workshop covering AI basics—though only once or twice." (N18)  "Training." (N20)  "Distribute instructional guides rather than formal courses—clinical workloads are already heavy." (N21)  "We participated in informatics training... to learn about AI integration." (N22)  "Hospitals should provide comprehensive AI training—system operations, data analysis, troubleshooting." (N23)  "Offer professional counseling for AI-related stress and challenges." (N23)  "Many nurses lack exposure to AI. If departments implement it, foundational training is necessary." (N26)  "Training is non-negotiable—most lack expertise. Combine instruction with hands-on practice." (N28)  "Regular workshops to identify effective AI tools and optimization strategies." (N29)  "Provide some training." (N31) |
